# Epidemiological Impact of SARS-CoV-2 Vaccination: Mathematical Modeling Analyses

**DOI:** 10.3390/vaccines8040668

**Published:** 2020-11-09

**Authors:** Monia Makhoul, Houssein H. Ayoub, Hiam Chemaitelly, Shaheen Seedat, Ghina R. Mumtaz, Sarah Al-Omari, Laith J. Abu-Raddad

**Affiliations:** 1 Infectious Disease Epidemiology Group, Weill Cornell Medicine-Qatar, Cornell University, Qatar Foundation—Education City, Doha 24144, Qatar; mom2039@qatar-med.cornell.edu (M.M.); hsc2001@qatar-med.cornell.edu (H.C.); shs4004@qatar-med.cornell.edu (S.S.); 2 World Health Organization Collaborating Centre for Disease Epidemiology Analytics on HIV/AIDS, Sexually Transmitted Infections, and Viral Hepatitis, Weill Cornell Medicine–Qatar, Cornell University, Qatar Foundation—Education City, Doha 24144, Qatar; 3 Department of Population Health Sciences, Weill Cornell Medicine, Cornell University, New York, NY 10022, USA; 4 Department of Mathematics, Statistics, and Physics, Qatar University, Doha 2713, Qatar; hayoub@qu.edu.qa; 5 Department of Epidemiology and Population Health, American University of Beirut, Beirut 11-0236, Lebanon; gm15@aub.edu.lb (G.R.M.); sia34@mail.aub.edu (S.A.-O.)

**Keywords:** SARS-CoV-2, COVID-19, coronavirus, epidemiology, vaccine, mathematical model

## Abstract

This study aims to inform SARS-CoV-2 vaccine development/licensure/decision-making/implementation, using mathematical modeling, by determining key preferred vaccine product characteristics and associated population-level impacts of a vaccine eliciting long-term protection. A prophylactic vaccine with efficacy against acquisition (*VE*_S_) ≥70% can eliminate the infection. A vaccine with *VE*_S_ <70% may still control the infection if it reduces infectiousness or infection duration among those vaccinated who acquire the infection, if it is supplemented with <20% reduction in contact rate, or if it is complemented with herd-immunity. At *VE*_S_ of 50%, the number of vaccinated persons needed to avert one infection is 2.4, and the number is 25.5 to avert one severe disease case, 33.2 to avert one critical disease case, and 65.1 to avert one death. The probability of a major outbreak is zero at *VE*_S_ ≥70% regardless of the number of virus introductions. However, an increase in social contact rate among those vaccinated (behavior compensation) can undermine vaccine impact. In addition to the reduction in infection acquisition, developers should assess the natural history and disease progression outcomes when evaluating vaccine impact.

## 1. Introduction

Following the Severe Acute Respiratory Syndrome (SARS) epidemic in 2002 and the Middle East Respiratory Syndrome (MERS) epidemic in 2012 [1], a novel coronavirus, the severe acute respiratory syndrome coronavirus 2 (SARS-CoV-2), emerged in late December 2019 in Wuhan, Hubei province, China [2,3]. While the earlier coronavirus epidemics were rather limited in scope and scale [1], SARS-CoV-2 rapidly spread [4] and evolved into a pandemic [5].

In the absence of an even partially efficacious vaccine [6], containment of the epidemic in China necessitated large-scale contact tracing and testing through the deployment of thousands of healthcare fieldworkers along with severe quarantine measures [4]. The strain put on healthcare systems [7] and the global human [8,9] and economic [10] losses caused by the virus and the resulting disease, designated as Coronavirus Disease 2019 (COVID-2019) [11], accelerated efforts towards vaccine development [6,12]. While multiple vaccine candidates are currently in the pipeline, they are still in the early stages of development [6,12,13].

Assessment of the population-level impact of vaccine candidates through mathematical modeling is a critical component in the process of vaccine development, value proposition, licensure, decision-making, and pathways and costs of vaccine administration and has been utilized for a wide range of infectious diseases [14,15,16,17,18,19,20,21,22,23,24,25,26,27,28]. In the early stages of development, modeling is used to define the vaccine’s key preferred product characteristics by estimating levels of efficacy necessary to observe a significant population-level impact, determining the necessary duration of protection/immunity incurred by the vaccine, and identifying priority populations for optimal effectiveness [21,29,30]. These parameters provide early guidance to developers, manufacturers, regulators, and decision-makers about candidates that are likely to be optimal through specifying vaccine characteristics that will maximize public health impact and cost-effectiveness [21,28,29,31,32]. Once key attributes are established, modeling plays an integral role in building the case for investment in vaccine development, and in ensuring rapid roll-out post-licensing, through assessment of risks, costs, and predicted returns associated with different immunization strategies [29,33]. Post-vaccination, modeling is used to inform the design and interpretation of surveillance studies [25,26,27].

We aimed in this study to provide the scientific evidence necessary to inform and accelerate SARS-CoV-2 vaccine development, licensure, decision-making, and implementation by determining key preferred vaccine product characteristics and associated population-level impact, at a critical time for such development [6,12,13]

## 2. Materials and Methods

### 2.1. Mathematical Model

A deterministic model was constructed to describe SARS-CoV-2 transmission dynamics in a given population, namely China as an illustrative example, in the presence of vaccination (Figure S1). The model extended a recently developed age-structured model focused on analyzing SARS-CoV-2 epidemiology in China [34]. The model’s structure was informed by current understanding of SARS-CoV-2′s natural history and epidemiology and consisted of a set of coupled nonlinear differential equations that stratified the population into compartments based on vaccination status, age group, infection status, infection stage, and disease stage (Text S1A). Vaccine impact was assessed over the course of one epidemic cycle, and the population was assumed to be stable with no births introduced. The developed model was informed by existing literature on the direct and indirect effects of vaccination such as vaccine impact on epidemic size, critical vaccination threshold, and effectiveness, assuming different types of vaccine efficacy [35,36,37,38,39,40]. The latter includes, in addition to the classical efficacy against susceptibility, efficacies against infectiousness and disease progression (Table 1) [35,36,37,38,39,40].

For both vaccinated and unvaccinated populations, nine age groups were considered, each representing a 10-year age band except for the last category (0–9, 10–19, …, ≥80 years). Susceptible individuals were at risk of being exposed to the infection at varying hazard rates depending on their age group and vaccination status. Following a latency period, infected individuals develop asymptomatic or mild infection followed by recovery, or severe infection followed by severe disease and then recovery, or critical infection followed by critical disease and either recovery or disease mortality. Mixing between individuals of different age groups was determined by an age-mixing matrix that allows a range of mixing (Text S1). The level of assortativeness in mixing between the different age groups was determined by a parameter whose value was set based on an earlier study that indicated limited assortativeness in mixing by age in the original Wuhan outbreak in China [34]. Details on model structure are in Supplementary Material. The model was coded, fitted, and analyzed using MATLAB R2019a [41].

### 2.2. Model Parameterization and Fitting

The model was parameterized and calibrated using empirical data on SARS-CoV-2′s natural history and epidemiology. Age-specific distributions of infected individuals across the mild, severe, or critical infection stages were based on case-severity levels observed in China [4,42,43]. Critical disease cases were at risk of disease mortality, with the relative mortality rate in each age group informed by the age-specific crude case fatality rate observed in China [3,44]. Population size, demographic structure (age distribution), and life expectancy, as of 2020, were obtained from the United Nations World Population Prospects database [45]. The model was fitted to empirical time series data for the daily and cumulative numbers of diagnosed SARS-CoV-2 cases and deaths [46], number of recovered individuals [46], and the age-specific attack rate [44,47]. Details of model parameters, values, and justifications are in Tables S1 and S2 and Text S1B,C.

### 2.3. Product Characteristics of Candidate Vaccines

We assessed the impact of a prophylactic vaccine that reduces susceptibility to infection. However, since the first available vaccine may only be partially efficacious against infection acquisition, we also assessed the impact of the vaccine assuming additional “breakthrough” effects, that is, effects that modulate the natural history of infection for those who are vaccinated but still acquire the infection. Specifically, we assumed that vaccination may reduce infectiousness per one contact (by reducing viral load), infection duration (by faster clearance with vaccine-induced immunity), and likelihood of developing severe or critical disease (by rapid immune response that prevents disease progression). Definitions of these vaccine efficacies are summarized in Table 1.

Other relevant characteristics include the duration of protection elicited by the vaccine and vaccination effect on adherence to social distancing; we investigated the impact of increasing social contact rate following vaccination with the perception of protection.

### 2.4. Measures of Vaccine Impact

The population-level impact of SARS-CoV-2 vaccination was assessed by quantifying incidence, cumulative incidence, and reduction in the incidence of infections, severe disease cases, critical disease cases, and deaths arising in the presence of vaccination compared to the counterfactual scenario of no-vaccination. Vaccination impact was further assessed by quantifying effectiveness, which is the number of vaccinated persons needed to avert one infection or one adverse disease outcome (ratio of number of vaccinations relative to that of averted outcomes). The latter measure is essentially cost-effectiveness, with no costs included as they are not yet available, and is therefore likely to be influential in informing vaccine prioritization to different segments of the population such as individuals in different age groups. Vaccination impact was assessed at: (1) $VE_{S}=50\%$ but $VE_{I}=VE_{P_{1}}=VE_{P_{2}}=0\%$, (2) $VE_{I}=50\%$ but $VE_{S}=VE_{P_{1}}=VE_{P_{2}}=0\%$, (3) $VE_{P_{1}}=50\%$ but $VE_{S}=VE_{I}=VE_{P_{2}}=0\%$, (4) $VE_{P_{2}}=50\%$ but $VE_{S}=VE_{I}=VE_{P_{1}}=0\%$, and (5) $VE_{S}=VE_{I}=VE_{P_{1}}=50\%$. Vaccine was assumed to elicit protection over 10 years, with this duration being exponentially distributed.

### 2.5. Vaccination Program Scenarios

Two vaccination program scenarios were considered. In both programs, it was assumed that vaccination is introduced in the absence of social-distancing interventions, as the purpose of vaccination is to replace such interventions. The first program scenario assumes vaccine introduction and scale-up to 80% coverage before epidemic onset. This scenario is relevant for assessing the impact of vaccination on future SARS-CoV-2 introductions in countries where the epidemic has been contained or at a low level, such as in China. The scenario is also relevant to assess the *maximum* potential impact of vaccination regardless of current epidemic status. The second program scenario assumes vaccine introduction during the epidemic’s exponential growth phase, with scale-up to 80% coverage within one month.

### 2.6. Additional Analyses

Incidence of new infections was assessed at various levels of $VE_{S}$ to determine the minimum efficacy needed to fully control the infection, that is, to reach a negligible incidence level (end of epidemic cycle), if not complete elimination. Incidence was also assessed in a scenario where vaccination was introduced with a social-distancing intervention to estimate the level of social distancing needed to complement vaccination to control the infection. Incidence was assessed in another scenario where those vaccinated increased their social contacts (behavior compensation), to assess consequences on vaccination impact. Lastly, we derived and estimated the likelihood of the occurrence of a major outbreak following infection introduction in a vaccinated but infection-free population (Text S1D).

In addition, two sensitivity analyses were conducted to assess vaccine effectiveness at varying levels of vaccine coverage and at high levels of assortativeness in age group mixing.

### 2.7. Uncertainty Analysis

A multivariable uncertainty analysis was conducted to determine the range of uncertainty around model predictions using five hundred model runs. At each run, Latin Hypercube sampling [48,49] was applied in selecting the natural history and disease progression parameter values from ranges specified by assuming ±30% uncertainty around parameters’ point estimates. The model was then refitted to input data and vaccine impact assessed in the new fitted model. The resulting distribution for vaccine impact across all 500 runs was used to calculate predicted means and 95% uncertainty intervals (UIs).

## 3. Results

**Figure 1:**
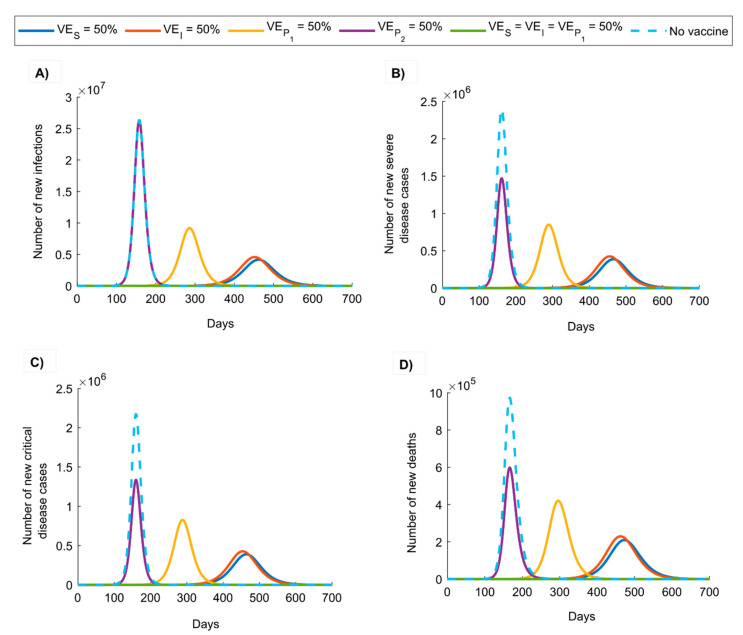
Impact of SARS-CoV-2 vaccination on the number of (**A**) new infections, (**B**) new severe disease cases, (**C**) new critical disease cases, and (**D**) new deaths in the scenario assuming vaccine scale-up to 80% coverage before epidemic onset. The duration of vaccine protection is 10 years. Impact was assessed at $VE_{S}=50\%$, $VE_{I}=50\%$, $VE_{P_{1}}=50\%$, $VE_{P_{2}}=50\%$, $VE_{S}=VE_{I}=VE_{P_{1}}=50\%$.

**Figure 2:**
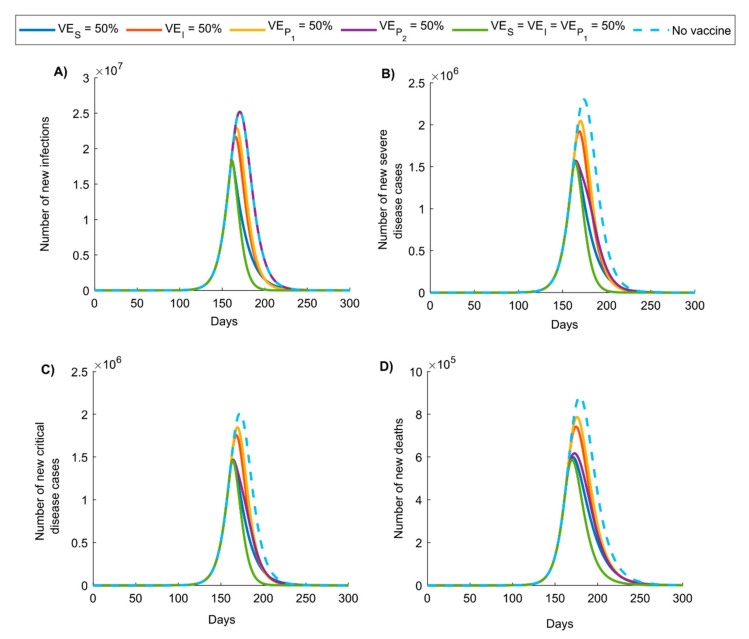
Impact of SARS-CoV-2 vaccination on the number of (**A**) new infections, (**B**) new severe disease cases, (**C**) new critical disease cases, and (**D**) new deaths in the scenario assuming vaccine introduction during the exponential growth phase of the epidemic, with scale-up to 80% coverage within one month. Duration of vaccine protection is 10 years. Impact was assessed at $VE_{S}=50\%$, $VE_{I}=50\%$, $VE_{P_{1}}=50\%$, $VE_{P_{2}}=50\%$, $VE_{S}=VE_{I}=VE_{P_{1}}=50\%$.

Figure 1 and Figure 2 illustrate the impact of vaccination assuming different vaccine product characteristics (efficacies; described in Table 1) for each vaccination program roll-out scenario (see Section 2 for details). In the first scenario (Figure 1 and Figures S2 and S3), where vaccination was scaled up to 80% coverage before epidemic onset, the epidemic in absence of vaccination peaked at 158 days after virus introduction but at 286 days when $VE_{P_{1}}=50\%$, 452 days when $VE_{I}=50\%$, and 462 days when $VE_{S}=50\%$. There was no epidemic when $VE_{S}=VE_{I}=VE_{P_{1}}=50\%$. A vaccine with $VE_{S}=50\%$ reduced peak infection incidence by 84.4% and cumulative/total infections by 52.8%, peak severe disease incidence by 83.9% and cumulative severe disease cases by 53.4%, peak critical disease incidence by 82.1% and cumulative critical disease cases by 46.7%, and peak death incidence by 79.0% and cumulative deaths by 44.4%. A vaccine with $VE_{I}=50\%$ yielded slightly lower reductions in the incidence of infection and adverse outcomes, while a vaccine with $VE_{P_{1}}=50\%$ was less impactful but still achieved considerable reductions. A vaccine with $VE_{P_{2}}=50\%$ had no impact on infection incidence, but reduced peak incidence of each of severe and critical disease by 38.5% and deaths by 40.0% (Figure 1), and the cumulative incidence of the latter three outcomes by ~39% (Figures S2 and S3).

In the second scenario (Figure 2 and Figures S4 and S5), where vaccination was rapidly scaled up to 80% coverage during the exponential growth phase, the epidemic peaked earlier and at lower values for incidence of infection and adverse outcomes. The impact of a vaccine with $VE_{S}=50\%$ was initially similar to that of a vaccine with $VE_{S}=VE_{I}=VE_{P_{1}}=50\%$; however, over time, the latter was more impactful in reducing infection and adverse outcomes. Reduction in the cumulative number of new infections (at end of epidemic cycle) was highest for $VE_{S}=VE_{I}=VE_{P_{1}}=50\%$ at 53.4%, followed by $VE_{S}=50\%$ at 41.2%, $VE_{I}=50\%$ at 28.2%, and $VE_{P_{1}}=50\%$ at 23.1%, with no reduction for $VE_{P_{2}}=50\%$. Reduction in cumulative number of new deaths for these efficacies was, respectively, 47.2%, 34.8%, 22.5%, 18.0%, and 30.0%.

**Figure 3:**
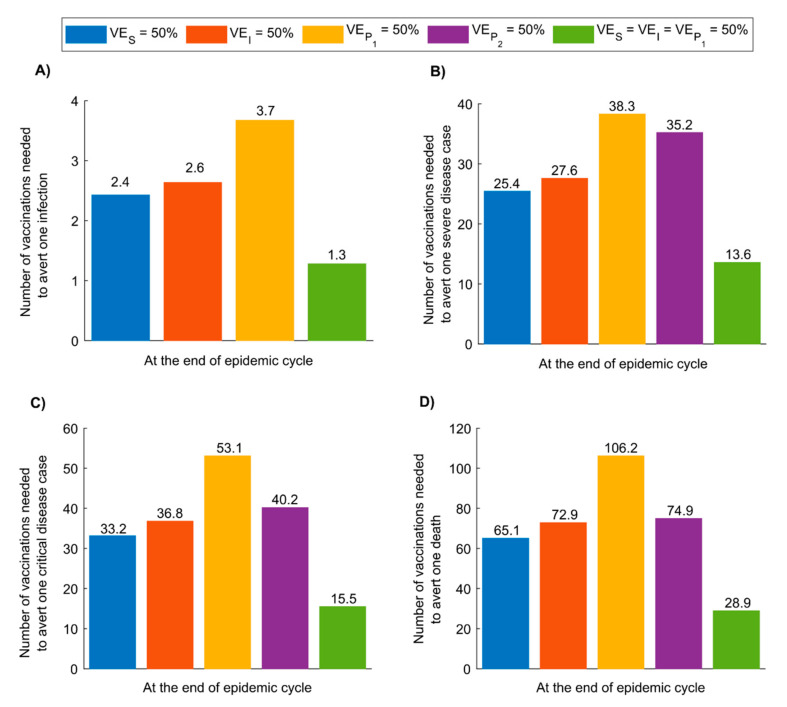
SARS-CoV-2 vaccine effectiveness. Number of vaccinated persons needed to avert (**A**) one infection, (**B**) one severe disease case, (**C**) one critical disease case, and (**D**) one death, by the end of the epidemic cycle, that is, after the epidemic has reached its peak and declined to a negligible level. The scenario assumes vaccine scale-up to 80% coverage before epidemic onset. Duration of vaccine protection is 10 years. Impact was assessed at $VE_{S}=50\%$, $VE_{I}=50\%$, $VE_{P_{1}}=50\%$, $VE_{P_{2}}=50\%$, $VE_{S}=VE_{I}=VE_{P_{1}}=50\%$. Panel A does not include the result for $VE_{P_{2}}=50\%$, as this efficacy has no impact on the number of infections—it affects only severe and critical disease and death.

Figure 3 illustrates vaccine effectiveness in averting infection and adverse outcomes by the end of the epidemic cycle (that is, after the epidemic has reached its peak and declined to a negligible level) for the first program scenario. For $VE_{S}=50\%$, 2.4 vaccinated persons were needed to avert one infection, 25.4 to avert one severe disease case, 33.2 to avert one critical disease case, and 65.1 to avert one death. Effectiveness was nearly comparable for $VE_{I}=50\%$, whereas more vaccinated persons were needed to avert one infection or one adverse outcome for $VE_{P_{1}}=50\%$ and $VE_{P_{2}}=50\%$. The best effectiveness was for $VE_{S}=VE_{I}=VE_{P_{1}}=50\%$ where only 1.3 vaccinated persons were needed to avert one infection, 13.6 to avert one severe disease case, 15.5 to avert one critical disease case, and 28.9 to avert one death. Graphs illustrating temporal evolution of vaccine effectiveness for vaccination program scenarios 1 and 2 are shown in Figures S6 and S7, respectively.

**Figure 4:**
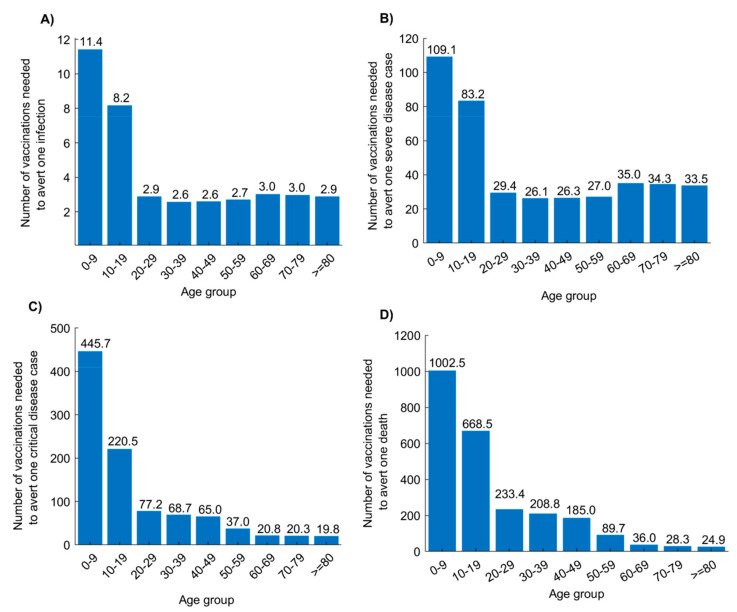
Effectiveness of age-group prioritization using a SARS-CoV-2 vaccine with *VE_s_* of 50%. Number of vaccinated persons needed to avert (**A**) one infection, (**B**) one severe disease case, (**C**) one critical disease case, and (**D**) one death by prioritizing different age groups for vaccination. Scenario assumes vaccine scale-up to 80% coverage before epidemic onset and duration of vaccine protection of 10 years. Effectiveness is assessed at the end of the epidemic cycle, that is, after the epidemic has reached its peak and declined to a negligible level.

Figure 4 shows the effectiveness of age-group prioritization by the end of the epidemic cycle using a vaccine introduced before epidemic onset with $VE_{S}=50\%$. Prioritizing adults ≥20 years of age was most effective in reducing infection incidence. By prioritizing each age group ≥20 years of age, ≤3 vaccinated persons were needed to avert one infection. By vaccinating the entire age bracket of those who are ≥20 years of age, 2.4 vaccinated persons were needed to avert one infection. Meanwhile, prioritizing adults ≥60 years of age was most effective in reducing new deaths, with ≤36 vaccinated persons needed to avert one death. Prioritizing children was least effective, with a large number of vaccinated persons needed to avert one infection or one adverse outcome. Note that there are minor differences in effectiveness over time. For instance, in the initial phases of the epidemic, prioritizing those 60–69 years of age was slightly more effective than prioritizing those 40–49 years of age (Figure S8). Meanwhile, towards the end of the epidemic cycle, the inverse was true.

**Figure 5:**
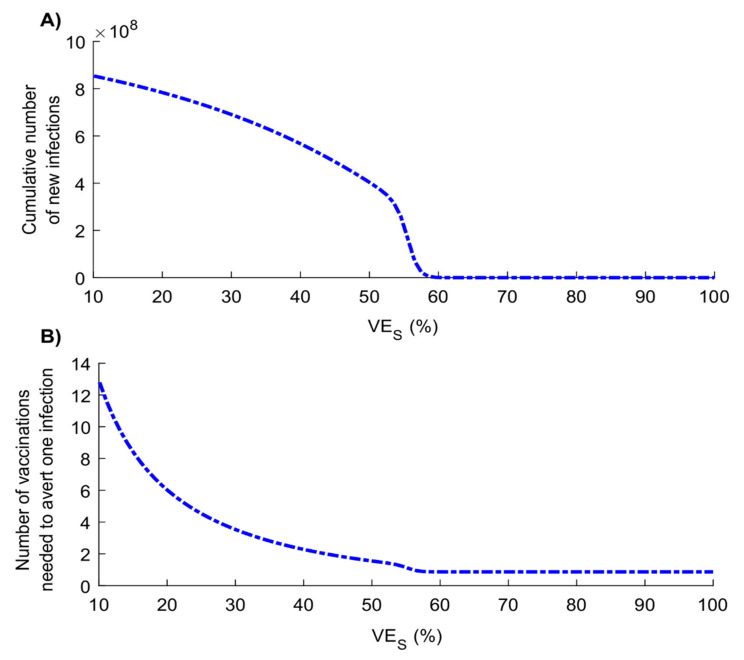
Impact of varying levels of vaccine efficacy in reducing susceptibility (*VE_s_*) on (**A**) cumulative number of new SARS-CoV-2 infections (final epidemic size) and (**B**) number of vaccinated persons needed to avert one SARS-CoV-2 infection. Scenario assumes vaccine scale-up to 80% coverage before epidemic onset. Duration of vaccine protection is 10 years. Measures are assessed at the end of the epidemic cycle, that is, after the epidemic has reached its peak and declined to a negligible level.

Figure 5 shows cumulative number of infections, which is the final epidemic size at the end of the epidemic cycle at various $VE_{S}$ levels for a vaccine introduced before epidemic onset. A gradual decrease is observed as $VE_{S}$ increases, with an accelerated reduction as $VE_{S}$ approaches 60%—the level beyond which the number of infections approaches zero. Epidemic onset is prevented at $VE_{S}=69\%$. Figure 5B illustrates the gains in effectiveness as $VE_{S}$ increases, with 5.3 vaccinated persons needed to avert one infection at $VE_{S}=30\%$, but only 1.3 at $VE_{S}=60\%$.

While a vaccine with $VE_{S}=50\%$ cannot fully control the epidemic, Figure S9 shows the impact when vaccination is supplemented with a social-distancing intervention that reduces the contact rate. A reduction in contact rate less than 20% would be sufficient to fully control the epidemic.

**Figure 6:**
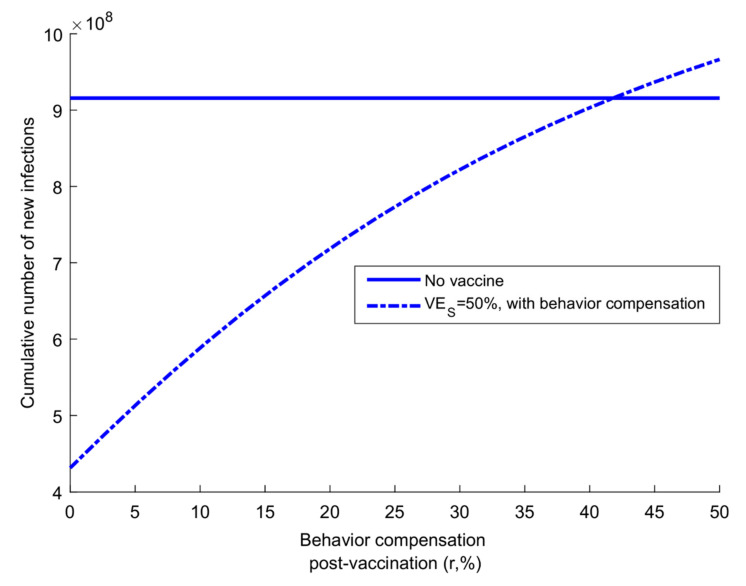
Impact of vaccination with reduced adherence to social distancing for those vaccinated. Figure shows the impact of varying levels of behavior compensation post-vaccination on the vaccine-induced reduction in the cumulative number of new SARS-CoV-2 infections by the end of the epidemic cycle. Scenario assumes vaccine scale-up to 80% coverage before epidemic onset, *VE_s_* is 50%, and duration of vaccine protection is 10 years.

Vaccinated individuals may increase their contact rate with the perception of protection. Figure 6 shows the consequences of behavior compensation. A 20% increase in contact rate among those vaccinated lowers the reduction in cumulative incidence from 52.8% to only 21.0%. A 41.8% increase in contact rate nullified the impact of vaccination in reducing incidence.

**Figure 7:**
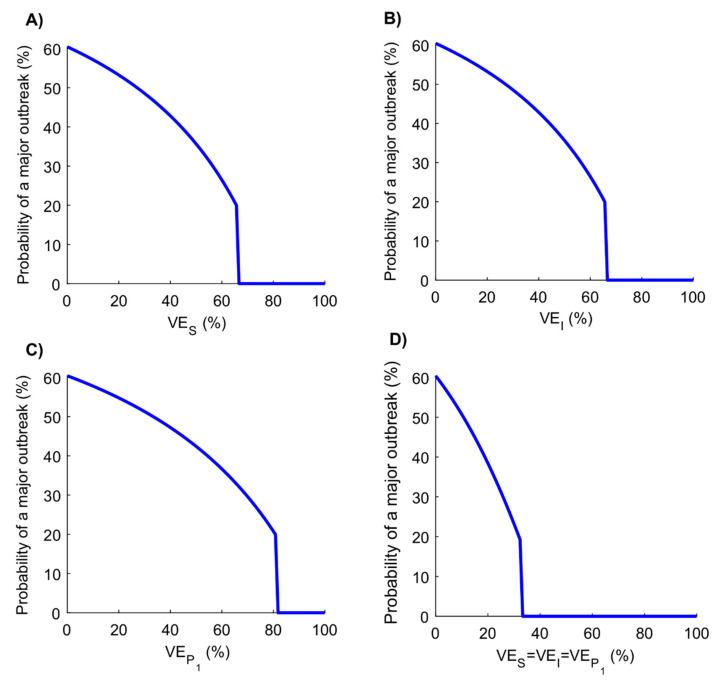
Probability of occurrence of a major outbreak following vaccination. Probability of occurrence of a major outbreak upon virus introduction at varying levels of (**A**) $VE_{S}$, (**B**) $VE_{I}$, (**C**) $VE_{P_{1}}$, and (**D**) $VE_{S}=VE_{I}=VE_{P_{1}}$. Scenario assumes vaccine scale-up to 80% coverage before epidemic onset. Duration of vaccine protection is 10 years. The figure does not include the result for $VE_{P_{2}}$, as this efficacy has no impact on the probability of occurrence of a major outbreak. The analysis and derivation for the probability of occurrence of a major outbreak can be found in Text S1D of the Supplementary Material.

Figure 7 reports the probability of occurrence of a major outbreak at varying levels of vaccine efficacy. A gradual decrease is noted with the increase in efficacy that accelerates close to $VE_{S}$ or $VE_{I}$ of ~70%, $VE_{P_{1}}$ of ~85%, and $VE_{S}=VE_{I}=VE_{P_{1}}$ of ~33%, beyond which no major outbreak is expected to occur. Note that this figure shows (conservatively) the upper bound of the probability of a major outbreak. Results for the lower bound are in Figure S10.

Our sensitivity analyses showed that effectiveness was not strongly dependent on vaccine coverage (Figure S11A), but that high assortativeness in age group mixing would result in considerably more vaccinations needed to avert one infection among children while not substantially affecting effectiveness in the older age groups (Figure S11B).

Uncertainty analysis demonstrated robustness of model predictions to a wide range of uncertainty in input parameters (Figure S12).

## 4. Discussion

The above results indicate that even a partially efficacious vaccine can offer a fundamental solution to the SARS-CoV-2 pandemic—the vaccine does not need to have sterilizing immunity to fully control the infection. Indeed, a vaccine with $VE_{S}$ ≥70% could be sufficient to control the pandemic at ≥80% coverage (Figure 5A). Even a vaccine with $VE_{S}$ <70% may still control the infection if it additionally (and plausibly) induces “breakthrough” effects such as reduction in viral load (reduction in infectiousness; $VE_{I}$) or faster infection clearance (reduction in infection duration; $VE_{P_{1}}$) among those vaccinated who still acquire the infection. The latter effects individually (that is, in absence of protection against acquisition) have a comparable impact on transmission to that of a prophylactic vaccine that reduces infection acquisition (Figure 1 and Figure 2). Even in the absence of such effects, infection control can still be achieved if vaccination is supplemented with only a moderate social-distancing intervention (Figure S9) or complemented with partial herd immunity—a considerable fraction of the population could have acquired the infection and developed protective antibodies by the time of vaccine roll-out. Even a vaccine that does not prevent infection yet only mitigates disease progression (reduction in severe or critical disease and death; $VE_{P_{2}}$) could still yield significant gains by curbing disease burden (Figure 1 and Figure 2).

Results also indicated that vaccine impact depends on the time of vaccine introduction, whether before (Figure 1 and Figures S2 and S3) or after (Figure 2 and Figures S4 and S5) epidemic onset and/or growth; maximal gains are achieved with earlier introduction. Early introduction optimally defers epidemic growth, flattens the incidence curve, and reduces the number of infections and disease outcomes (Figure 1, Figure 2 and Figures S2–S5).

The vaccine will likely be cost-effective over a broad range of efficacy levels (Figure 5B). For a vaccine with $VE_{S}$ of 50%, the number of vaccinations needed to avert one infection is only 2.4, 25.5 are needed to avert one severe disease case is, 33.2 to avert one critical disease case, and 65.1 to avert one death (Figure 3). Return on effectiveness is also rapid for such a respiratory infection with a fast-growing epidemic scale (Figures S6 and S7).

Effectiveness can be further enhanced by prioritizing vaccination for those ≥60 years of age for an optimal reduction in disease cases and deaths (Figure 4). Conversely, prioritizing children is least effective, with their lower risk for developing adverse outcomes [4,50,51] (Figure 4). This being said, prioritizing vaccination for any single age group, regardless of age group, has overall lower effectiveness than extending vaccination to all age groups—vaccinating only one age group reduces the reproduction number ($R_{0}$) only marginally, whereas vaccinating all age groups reduces $R_{0}$ to an epidemic domain where small reductions in $R_{0}$ can have a more substantial impact on epidemic size (Figure S13, also Figure 3 versus Figure 4). Consequently, roll-out strategies should initially prioritize individuals ≥60 years of age but then incrementally cover younger age cohorts and eventually the entire population.

Vaccination will also reduce the likelihood of a major outbreak following virus introduction/reintroduction into a population (Figure 7). With a vaccine of $VE_{S}$ ≥70%, infection transmission chains may not be sustainable, regardless of the number of virus introductions. Of concern, however, is the potential increase in social contact rate among those vaccinated (behavior compensation): a 42% increase in contact rate can virtually nullify the gains of a vaccine with $VE_{S}$ of 50% at a coverage of 80% (Figure 6). Roll-out of a vaccine with intermediate efficacy should be coupled with public health communication that stresses caution in social mixing following vaccination.

This study has limitations. Model estimations are contingent on the validity and generalizability of input data. While we used available evidence for SARS-CoV-2 natural history and epidemiology, our understanding of its epidemiology is still evolving. We assessed vaccine impact using China as an illustrative example, where the outbreak first emerged, yet evidence suggests that many infections may have been undocumented in this country, particularly in the early epidemic phase [52]. This may affect some estimates, such as mortality, probably towards overestimation [34,53]. While the absolute impact of the vaccine on disease severity and mortality may have been overestimated, the relative impact (reduction rate) is less likely to have been affected. Our baseline $R_{0}$ for China was 2.1 [34], but $R_{0}$ may vary across settings as suggested by existing evidence [54,55], thus affecting estimates for the minimum efficacy or the minimum coverage needed for infection elimination. For instance, for an $R_{0}$ of 3, the minimum $VE_{S}$ needed for elimination is about 90% (Figure S14). Similarly, higher vaccine coverage levels will be needed to achieve elimination at higher values of $R_{0}$. We assessed vaccine impact in a country with limited infection spread relative to population size, but future work will need to factor differences in the epidemic phase in modeling assessments of vaccine impact in other countries. We also assessed vaccine impact for one epidemic cycle, with no assessment of seasonality or future cycles. Assessment of the long-term impact of vaccination will require an extension of the model to factor in the waning of natural immunity among recovered individuals; rapid waning of natural immunity or vaccine immunity will require more and repeat vaccinations. We assumed a long duration of vaccine protection (10 years), but this has limited impact on the predictions for one epidemic cycle, provided the duration of vaccine protection is greater than one year. Despite these limitations, our model was complex enough to factor the different key vaccine product characteristics but also parsimonious enough to be tailored to the nature of available data. The model also generated results that are valid to a wide range of model assumptions.

## 5. Conclusions

With most of the world’s population remaining susceptible to SARS-CoV-2 and the need to impose disruptive social-distancing interventions, vaccination is a reliable intervention in the long term. Findings show that even a partially efficacious vaccine provides a fundamental solution to the SARS-CoV-2 pandemic and at high cost-effectiveness. Vaccine impact and cost-effectiveness will not only depend on its efficacy in preventing infection but can be enhanced if those vaccinated who still acquire the infection have reduced infectiousness, duration of infection, and disease severity. Vaccine developers should thus not only assess the primary endpoint of reduction in acquisition but also other outcomes and/or proxy biomarkers including reductions in viral load and disease outcomes and speed of infection clearance for those vaccinated and unvaccinated. The totality of these primary and secondary endpoints may prove critical in the licensure process, decision-making, and vaccine impact once introduced into a population.

## Figures and Tables

**Table 1:** Key vaccine product characteristics used to assess impact of a vaccine against SARS-CoV-2.

Vaccine Characteristic	Definition	Description
$VE_{S}$	Vaccine efficacy in reducing susceptibility	Proportional reduction in the susceptibility to infection acquisition among those vaccinated compared to those unvaccinated
$VE_{I}$	Vaccine efficacy in reducing infectiousness	Proportional reduction in infectiousness (lower viral load due to vaccine-primed immune response) among those who are vaccinated but acquire the infection compared to those unvaccinated
$VE_{P_{1}}$	Vaccine efficacy in reducing the duration of infection	Proportional reduction in the duration of mild infection (faster infection clearance due to vaccine-primed immune response) among those who are vaccinated but still acquire the infection compared to those unvaccinated
$VE_{P_{2}}$	Vaccine efficacy in reducing the fraction of individuals with severe or critical infection	Proportional reduction in the fraction of individuals with severe or critical infection (lower probability of developing severe or critical infection due to vaccine-primed immune response) among those who are vaccinated but still acquire the infection compared to those unvaccinated
D	Duration of vaccine protection	Duration of protection that the vaccine will elicit
r	Behavior compensation post-vaccination	Proportional increase in social contact rate (reduced social distancing) among those who are vaccinated compared to those unvaccinated

## Supplementary Materials

The following are available online at https://www.mdpi.com/2076-393X/8/4/668/s1, Text S1A: Model structure, Text S1B: Parameter values, Text S1C: The basic reproduction number *R*_0_, Text S1D: Probability of a major outbreak. Figure S1: Schematic diagram describing the basic structure of the SARS-CoV-2 vaccine model, Figure S2: Impact of SARS-CoV-2 vaccination on the cumulative number of (A) new infections, (B) new severe disease cases, (C) new critical disease cases, and (D) new deaths in the scenario assuming vaccine scale-up to 80% coverage before epidemic onset, Figure S3: Role of SARS-CoV-2 vaccination in reducing the cumulative number of (A) new infections, (B) new severe disease cases, (C) new critical disease cases, and (D) new deaths in the scenario assuming vaccine scale-up to 80% coverage before epidemic onset, Figure S4: Impact of SARS-CoV-2 vaccination on the cumulative number of (A) new infections, (B) new severe disease cases, (C) new critical disease cases, and (D) new deaths in the scenario assuming vaccine introduction during the exponential growth phase of the epidemic, with scale-up to 80% coverage within one month, Figure S5: Role of SARS-CoV-2 vaccination in reducing the cumulative number of (A) new infections, (B) new severe disease cases, (C) new critical disease cases, and (D) new deaths in the scenario assuming vaccine introduction during the exponential growth phase of the epidemic, with scale-up to 80% coverage within one month, Figure S6: Temporal evolution of SARS-CoV-2 vaccine effectiveness in the scenario assuming vaccine scale-up to 80% coverage before epidemic onset, Figure S7: Temporal evolution of SARS-CoV-2 vaccine effectiveness in the scenario assuming vaccine introduction during the exponential growth phase of the epidemic, with scale-up to 80% coverage within one month, Figure S8: Temporal evolution of effectiveness of age-group prioritization using a SARS-CoV-2 vaccine with *VE*_S_ of 50%, Figure S9: Impact of a social-distancing intervention reducing the contact rate in the population on the cumulative number of new SARS-CoV-2 infections, when introduced to supplement the impact of a vaccine that has 50% efficacy in reducing susceptibility, *VE*_S_, Figure S10: Probability of occurrence of a major outbreak following vaccination, Figure S11: Sensitivity analyses assessing vaccine effectiveness (number of vaccinated persons needed to avert one infection) at (A) varying levels of vaccine coverage and (B) high levels of assortativeness in age group mixing, Figure S12: Uncertainty analysis, Figure S13: Vaccine effectiveness of age-group prioritization and the reproduction number *R*_0_, Figure S14: Impact of varying levels of vaccine efficacy in reducing susceptibility, *VE*_S_, on the cumulative number of new SARS-CoV-2 infections when the reproduction number R0 is 3, Table S1: Definitions of population variables and symbols used in the model, Table S2: Model assumptions in terms of parameter values.

## Data and Materials Availability

All data are available within the manuscript and its supplementary materials. The codes programmed in MATLAB can be obtained by contacting the authors.

## One Sentence Summary

A partially efficacious vaccine with efficacy against acquisition (*VE*_S_) ≥70% at ≥80% coverage could be sufficient to control the pandemic with high cost-effectiveness.
