# Supplementary material for: Epidemiological Impact of SARS-CoV-2 Vaccination: Mathematical Modeling Analyses
Source: Vaccines (Basel). 2020 Nov 9;8(4):668. doi: 10.3390/vaccines8040668 (PMC7712303; doi:10.3390/vaccines8040668)
Supplement: Supplementary file 1 [file vaccines-08-00668-s001.pdf]

Supplementary Material

Epidemiological impact of SARS-CoV-2 vaccination: mathematical modeling analyses

Monia Makhoul[1,2,3†], Houssein H. Ayoub[4†], Hiam Chemaitelly[1,2], Shaheen Seedat[1,2,3], Ghina R. Mumtaz[5], Sarah Al-Omari[5], and Laith J. Abu-Raddad[1,2,3*]

[1]Infectious Disease Epidemiology Group, Weill Cornell Medicine-Qatar, Cornell University, Qatar Foundation - Education City, Doha, Qatar

[2]World Health Organization Collaborating Centre for Disease Epidemiology Analytics on HIV/AIDS, Sexually Transmitted Infections, and Viral Hepatitis, Weill Cornell Medicine–Qatar, Cornell University, Qatar Foundation – Education City, Doha, Qatar

[3]Department of Population Health Sciences, Weill Cornell Medicine, Cornell University, New York City, New York, USA

[4]Department of Mathematics, Statistics, and Physics, Qatar University, Doha, Qatar

[5]Department of Epidemiology and Population Health, American University of Beirut, Beirut, Lebanon

[*]To whom correspondence should be addressed: Email: lja2002@qatar-med.cornell.edu
[†]These authors contributed equally to the work.

Table of Content

Text S1A. Model structure.

Figure S1. Schematic diagram describing the basic structure of the SARS-CoV-2 vaccine model.

Table S1. Definitions of population variables and symbols used in the model.

Text S1B. Parameter values.

Text S1C. The basic reproduction number R_0.

Text S1D. Probability of a major outbreak.

Figure S2. Impact of SARS-CoV-2 vaccination on the cumulative number of A) new infections, B) new severe disease cases, C) new critical disease cases, and D) new deaths in the scenario assuming vaccine scale-up to 80% coverage before epidemic onset.

Figure S3. Role of SARS-CoV-2 vaccination in reducing the cumulative number of A) new infections, B) new severe disease cases, C) new critical disease cases, and D) new deaths in the scenario assuming vaccine scale-up to 80% coverage before epidemic onset.

Figure S4. Impact of SARS-CoV-2 vaccination on the cumulative number of A) new infections, B) new severe disease cases, C) new critical disease cases, and D) new deaths in the scenario assuming vaccine introduction during the exponential growth phase of the epidemic, with scale-up to 80% coverage within one month.

Figure S5. Role of SARS-CoV-2 vaccination in reducing the cumulative number of A) new infections, B) new severe disease cases, C) new critical disease cases, and D) new deaths in the scenario assuming vaccine introduction during the exponential growth phase of the epidemic, with scale-up to 80% coverage within one month.

Figure S6. Temporal evolution of SARS-CoV-2 vaccine effectiveness in the scenario assuming vaccine scale-up to 80% coverage before epidemic onset.

Figure S7. Temporal evolution of SARS-CoV-2 vaccine effectiveness in the scenario assuming vaccine introduction during the exponential growth phase of the epidemic, with scale-up to 80% coverage within one month.

Figure S8. Temporal evolution of effectiveness of age-group prioritization using a SARS-CoV-2 vaccine with VE_S of 50%.

Figure S9. Impact of a social-distancing intervention reducing the contact rate in the population on the cumulative number of new SARS-CoV-2 infections, when introduced to supplement the impact of a vaccine that has 50% efficacy in reducing susceptibility, VE_S.

Figure S10. Probability of occurrence of a major outbreak following vaccination.

Figure S11. Sensitivity analyses assessing vaccine effectiveness (number of vaccinated persons needed to avert one infection) at A) varying levels of vaccine coverage and B) high levels of assortativeness in age group mixing.

Figure S12. Uncertainty analysis.

Figure S13. Vaccine effectiveness of age-group prioritization and the reproduction number R_0.

Figure S14. Impact of varying levels of vaccine efficacy in reducing susceptibility, VE_S, on the cumulative number of new SARS-CoV-2 infections when the reproduction number R_0 is 3.

Text S1. SARS-CoV-2 mathematical vaccine model

A. Model structure

We extended a recently-developed age-structured deterministic compartmental model to describe the impact of vaccination on severe acute respiratory syndrome coronavirus 2 (SARS-CoV-2) transmission dynamics and progression of the resulting disease, Coronavirus Disease 2019 (COVID-2019), in a given population. The model stratifies the unvaccinated and vaccinated populations into compartments according to age group (0-9, 10-19, 20-29,…, ≥80 years), infection status (uninfected, infected), infection stage (mild, severe, critical), and disease stage (severe, critical). Transmission and disease progression dynamics in vaccinated and unvaccinated cohorts are described using age-specific sets of nonlinear ordinary differential equations, where each age group a ($a = 1, 2, ...9$) refers to a 10-year age band (0-9,10-19,..70-79) apart from the last group including all those aged ≥ 80 years. The model is illustrated in Figure S1.

Figure S1. Schematic diagram describing the basic structure of the SARS-CoV-2 vaccine model.

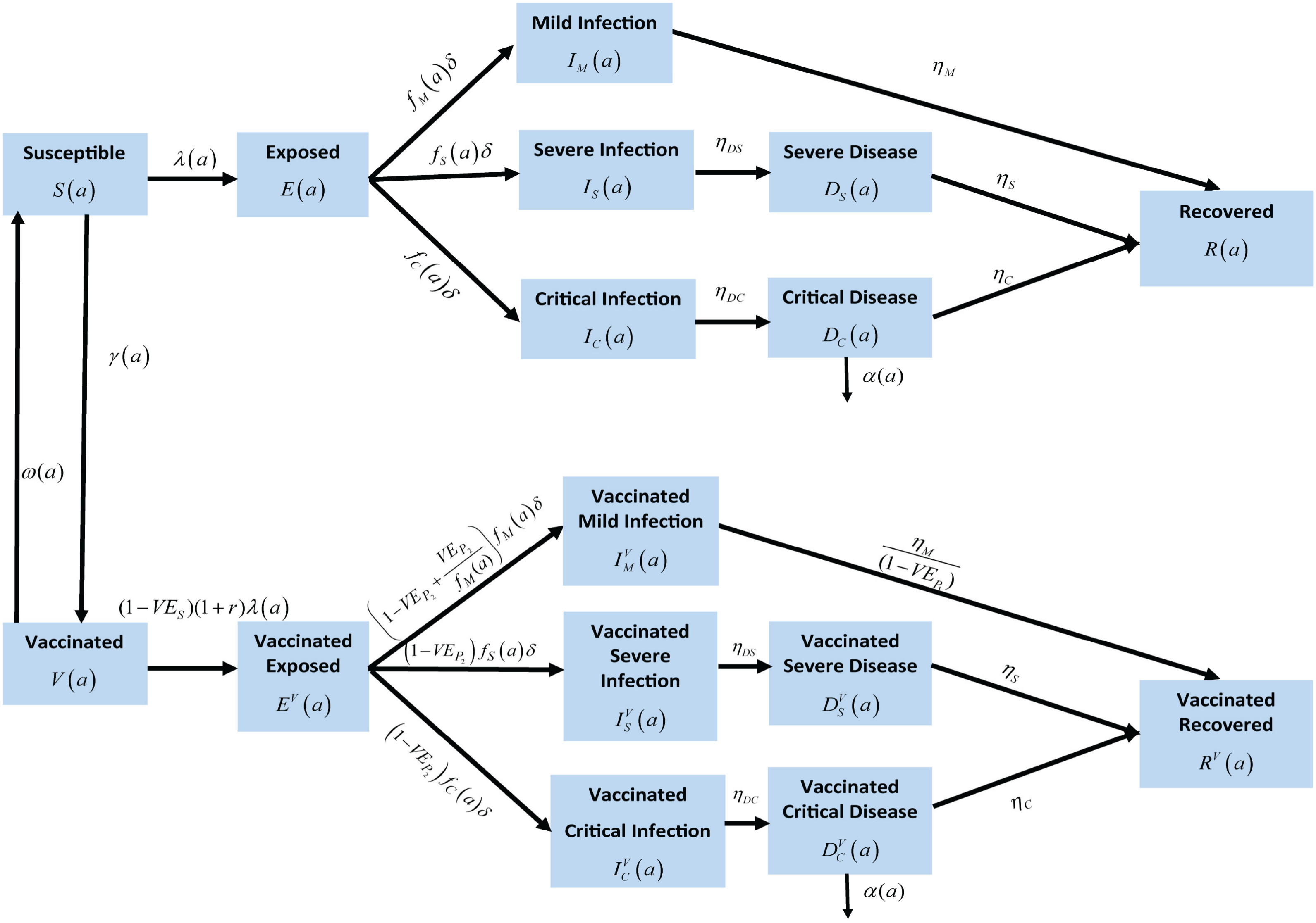

The following set of equations describes the transmission dynamics among unvaccinated and vaccinated populations in the first age group:

Unvaccinated population aged 0-9 years:

$$\frac{dS(1)}{dt} = -\left(\lambda(1) + \mu + \xi(1) - \gamma(1)\right)S(1) + \omega(1)V(1)$$

$$\frac{dE(1)}{dt} = \lambda(1)S(1) - \left(\delta + \mu + \xi(1)\right)E(1)$$

$$\frac{dI_M(1)}{dt} = f_M(1)\delta E(1) - \left(\eta_M + \mu + \xi(1)\right)I_M(1)$$

$$\frac{dI_S(1)}{dt} = f_S(1)\delta E(1) - \left(\eta_{DS} + \mu + \xi(1)\right)I_S(1)$$

$$\frac{dI_C(1)}{dt} = f_C(1)\delta E(1) - \left(\eta_{DC} + \mu + \xi(1)\right)I_C(1)$$

$$\frac{dD_S(1)}{dt} = \eta_{DS}I_S(1) - \left(\eta_S + \mu + \xi(1)\right)D_S(1)$$

$$\frac{dD_C(1)}{dt} = \eta_{DC}I_C(1) - \left(\eta_C + \mu + \xi(1) + \alpha(1)\right)D_C(1)$$

$$\frac{dR(1)}{dt} = \eta_M I_M(1) + \eta_S D_S(1) + \eta_C D_C(1) - \left(\mu + \xi(1)\right)R(1)$$

Vaccinated population aged 0-9 years:

$$\frac{dV(1)}{dt} = \gamma(1)S(1) - \left(\left(1 - VE_S\right)(1 + r)\lambda(1) + \mu + \xi(1) - \omega(1)\right)V(1)$$

$$\frac{dE^V(1)}{dt} = \left(1 - VE_S\right)(1 + r)\lambda(1)V(1) - \left(\delta + \mu + \xi(1)\right)E^V(1)$$

$$\frac{dI_M^V(1)}{dt} = \left((1 - VE_{P_2}) + \frac{VE_{P_2}}{f_M(1)}\right)f_M(1)\delta E^V(1) - \left(\frac{\eta_M}{\left(1 - VE_{P_1}\right)} + \mu + \xi(1)\right)I_M^V(1)$$

$$\frac{dI_S^V(1)}{dt} = \left(1 - VE_{P_2}\right)f_S(1)\delta E^V(1) - \left(\eta_{DS} + \mu + \xi(1)\right)I_S^V(1)$$

$$\frac{dI_C^V(1)}{dt} = \left(1 - VE_{P_2}\right)f_C(1)\delta E^V(1) - \left(\eta_{DC} + \mu + \xi(1)\right)I_C^V(1)$$

$$\frac{dD_S^V(1)}{dt} = \eta_{DS}I_S^V(1) - \left(\eta_S + \mu + \xi(1)\right)D_S^V(1)$$

$$\frac{dD_C^V(1)}{dt} = \eta_{DC}I_C^V(1) - \left(\eta_C + \mu + \xi(1) + \alpha(1)\right)D_C^V(1)$$

$$\frac{dR^V(1)}{dt} = \frac{\eta_M}{\left(1-VE_{P_1}\right)} I_M^V(1) + \eta_S D_S^V(1) + \eta_C D_C^V(1) - \left(\mu + \xi(1)\right) R^V(1)$$

For subsequent age groups, the following set of equations was used:

Unvaccinated populations aged 10+ years:

$$\frac{dS(a)}{dt} = \xi(a-1)S(a-1) - \left(\lambda(a) + \mu + \xi(a) - \gamma(a)\right)S(a) + \omega(a)V(a)$$

$$\frac{dE(a)}{dt} = \xi(a-1)E(a-1) + \lambda(a)S(a) - \left(\delta + \mu + \xi(a)\right)E(a)$$

$$\frac{dI_M(a)}{dt} = \xi(a-1)I_M(a-1) + f_M(a)\delta E(a) - \left(\eta_M + \mu + \xi(a)\right)I_M(a)$$

$$\frac{dI_S(a)}{dt} = \xi(a-1)I_S(a-1) + f_S(a)\delta E(a) - \left(\eta_{DS} + \mu + \xi(a)\right)I_S(a)$$

$$\frac{dI_C(a)}{dt} = \xi(a-1)I_C(a-1) + f_C(a)\delta E(a) - \left(\eta_{DC} + \mu + \xi(a)\right)I_C(a)$$

$$\frac{dD_S(a)}{dt} = \xi(a-1)D_S(a-1) + \eta_{DS}I_S(a) - \left(\eta_S + \mu + \xi(a)\right)D_S(a)$$

$$\frac{dD_C(a)}{dt} = \xi(a-1)D_C(a-1) + \eta_{DC}I_C(a) - \left(\eta_C + \mu + \xi(a) + \alpha(a)\right)D_C(a)$$

$$\frac{dR(a)}{dt} = \xi(a-1)R(a-1) + \eta_M I_M(a) + \eta_S D_S(a) + \eta_C D_C(a) - \left(\mu + \xi(a)\right)R(a)$$

Vaccinated populations aged 10+ years:

$$\frac{dV(a)}{dt} = \gamma(a)S(a) + \xi(a-1)V(a-1) - \left(\left(1-VE_S\right)\left(1+r\right)\lambda(a) + \mu + \xi(a) - \omega(a)\right)V(a)$$

$$\frac{dE^V(a)}{dt} = \xi(a-1)E^V(a-1) + \left(1-VE_S\right)\left(1+r\right)\lambda(a)V(a) - \left(\delta + \mu + \xi(a)\right)E^V(a)$$

$$\frac{dI_M^V(a)}{dt} = \xi(a-1)I_M^V(a-1) + \left((1-VE_{P_2}) + \frac{VE_{P_2}}{f_M(a)}\right)f_M(a)\delta E^V(a) - \left(\frac{\eta_M}{\left(1-VE_{P_1}\right)} + \mu + \xi(a)\right)I_M^V(a)$$

$$\frac{dI_S^V(a)}{dt} = \xi(a-1)I_S^V(a-1) + \left(1-VE_{P_2}\right)f_S(a)\delta E^V(a) - \left(\eta_{DS} + \mu + \xi(a)\right)I_S^V(a)$$

$$\frac{dI_C^V(a)}{dt} = \xi(a-1)I_C^V(a-1) + \left(1-VE_{P_2}\right)f_C(a)\delta E^V(a) - \left(\eta_{DC} + \mu + \xi(a)\right)I_C^V(a)$$

$$\frac{dD_S^V(a)}{dt} = \xi(a-1)D_S^V(a-1) + \eta_{DS}I_S^V(a) - \left(\eta_S + \mu + \xi(a)\right)D_S^V(a)$$

$$\frac{dD_C^V(a)}{dt} = \xi(a-1)D_C^V(a-1) + \eta_{DC}I_C^V(a) - \left(\eta_C + \mu + \xi(a) + \alpha(a)\right)D_C^V(a)$$

$$\frac{dR^V(a)}{dt} = \xi(a-1)R^V(a-1) + \frac{\eta_M}{(1-VE_{P_1})} I_M^V(a) + \eta_S D_S^V(a) + \eta_C D_C^V(a) - (\mu + \xi(a)) R^V(a)$$

The definitions of population variables and symbols used in the equations are in Table S1.

Table S1. Definitions of population variables and symbols used in the model.

Symbol	Definition
**Transmission dynamics parameters**	
$S(a)$	Unvaccinated susceptible population
$E(a)$	Unvaccinated latently infected population
$I_M(a)$	Unvaccinated population with asymptomatic or mild infection
$I_S(a)$	Unvaccinated population with severe infection
$I_C(a)$	Unvaccinated population with critical infection
$I(a)$	Unvaccinated infected population
$D_S(a)$	Unvaccinated population with severe disease
$D_C(a)$	Unvaccinated population with critical disease
$R(a)$	Unvaccinated recovered population
$V(a)$	Vaccinated susceptible population
$E^V(a)$	Vaccinated latently infected population
$I_M^V(a)$	Vaccinated population with asymptomatic or mild infection
$I_S^V(a)$	Vaccinated population with severe infection
$I_C^V(a)$	Vaccinated population with critical infection
$I^V(a)$	Vaccinated infected population
$D_S^V(a)$	Vaccinated population with severe disease
$D_C^V(a)$	Vaccinated population with critical disease
$R^V(a)$	Vaccinated recovered population
N	Total population size
n_{age}	Number of age groups
$\xi(a)$	Transition rate from one age group to the next age group
$\sigma(a)$	Susceptibility profile to the infection in each age group
β	Overall infectious contact rate
$1/\delta$	Duration of latent infection
$1/\eta_M$	Duration of asymptomatic or mild infection
$1/\eta_{DS}$	Duration of severe infection infectiousness before isolation and/or hospitalization
$1/\eta_S$	Duration of severe disease following onset of severe disease
$1/\eta_{DC}$	Duration of critical infection infectiousness before isolation and/or hospitalization

$1/\eta_C$	Duration of critical disease following onset of critical disease
$1/\mu$	Natural death rate
$CFR(a)$	*Relative* case fatality rate in each age group
$\alpha(a)$	Mortality rate in each age group
$f_M(a)$	Proportion of infections that will progress to be mild or asymptomatic infections
$f_S(a)$	Proportion of infections that will progress to be severe infections
$f_C(a)$	Proportion of infections that will progress to be critical infections
**Key vaccine product characteristics**	
VE_s	Vaccine efficacy in reducing susceptibility
VE_I	Vaccine efficacy in reducing infectiousness
VE_{P1}	Vaccine efficacy in reducing the duration of infection
VE_{P2}	Vaccine efficacy in reducing the fraction of individuals with severe or critical infection
$1/\omega$	Duration of vaccine protection
r	Behavior compensation post-vaccination

The force of infection (hazard rate of infection) experienced by the unvaccinated susceptible populations $S(a)$ is given by

$$\lambda(a)=\beta\sigma(a)\sum_{a'=1}^{n_{age}}\mathcal{H}_{a,a'}\frac{I(a')+(1-VE_I)I^V(a')}{\begin{bmatrix}S(a')+E(a')+I_M(a')+I_S(a')+I_C(a')+D_S(a')+D_C(a')+R(a')+\\V(a')+E^V(a')+I_M^V(a')+I_S^V(a')+I_C^V(a')+D_S^V(a')+D_C^V(a')+R^V(a')\end{bmatrix}},$$

while that of vaccinated susceptible populations $V(a)$ is given by

$$\lambda^V(a)=(1-VE_S)(1+r)\lambda(a)$$

where β is the overall infectious contact rate. The mixing among the different age groups is dictated by the mixing matrix $\mathcal{H}_{a,a''}$. This matrix provides the probability that an individual in the a age group will mix with an individual in the a' age group (regardless of vaccination status). The mixing matrix is given by

$$\mathcal{H}_{a,a'} = e_{Age}\delta_{a,a'} + (1-e_{Age})\frac{\begin{bmatrix} S(a')+E(a')+I_M(a')+I_S(a')+I_C(a')+D_S(a')+D_C(a')+R(a')+ \\ V(a')+E^V(a')+I_M^V(a')+I_S^V(a')+I_C^V(a')+D_S^V(a')+D_C^V(a')+R^V(a') \end{bmatrix}}{\sum_{a=1}^{n_{age}}\begin{bmatrix} S(a)+E(a)+I_M(a)+I_S(a)+I_C(a)+D_S(a)+D_C(a)+R(a)+ \\ V(a)+E^V(a)+I_M^V(a)+I_S^V(a)+I_C^V(a)+D_S^V(a)+D_C^V(a)+R^V(a) \end{bmatrix}}$$

Here, $\delta_{a,a'}$ is the identity matrix. $e_{Age} \in [0,1]$ measures the degree of assortativeness in the mixing. At the extreme $e_{Age} = 0$, the mixing is fully proportional. Meanwhile, at the other extreme, $e_{Age} = 1$, the mixing is fully assortative, that is individuals mix only with members in their own age group.

B. Parameter values

The input parameters of the model were chosen based on current empirical data for SARS-CoV-2 natural history and epidemiology. The parameter values are listed in Table S2.

Table S2. Model assumptions in terms of parameter values.

Parameter	Symbol	Value	Justification
Duration of latent infection	$1/\delta$	3.69 days	Based on existing estimate [1] and based on a median incubation period of 5.1 days [2] adjusted by observed viral load among infected persons [3] and reported transmission before onset of symptoms [4]
Duration of infectiousness	$1/\eta_M$; $1/\eta_{DS}$; $1/\eta_{DC}$	3.48 days	Based on existing estimate [1] and based on observed time to recovery among persons with mild infection [1, 5] and observed viral load in infected persons [3, 4]
Duration of severe disease following onset of severe disease	$1/\eta_S$	28 days	Observed duration from onset of severe disease to recovery [5]
Duration of hospitalization for critical infection	$1/\eta_C$	28 days	Observed duration from onset of critical disease to recovery [5]
Life expectancy in China	$1/\mu$	77.47 years	United Nations World Population Prospects database [6]
Relative case fatality rate in each age group	$CFR(a)$		Observed crude case fatality rate based on China data [7, 8]
Age 0-9 years		0%	
Age 10-39 years		0.2%	
Age 40-49 years		0.4%	
Age 50-59 years		1.3%	
Age 60-69 years		3.6%	
Age 70-79 years		8.0%	
Age 80+ years		21.9%	

Proportion of infections that will progress to be mild or asymptomatic infections	$f_M(a)$		Observed proportion of infections that eventually develop mild or asymptomatic in China [5, 9, 10]
Age 0-9 years		88.9%	
Age 10-49 years		88.0%	
Age 50-59 years		82.5%	
Age 60+ years		71.2%	
Proportion of infections that will progress to be severe infections	$f_S(a)$		Observed proportion of infections that eventually develop severe disease in China [5, 9, 10]
Age 0-9 years		11.1%	
Age 10-49 years		9.9%	
Age 50-59 years		10.3%	
Age 60+ years		7.8%	
Proportion of infections that will progress to be critical infections	$f_C(a)$		Observed proportion of infections that eventually develop critical disease in China [5, 9, 10]
Age 0-9 years		0.0%	
Age 10-49 years		2.2%	
Age 50-59 years		7.2%	
Age 60+ years		20.9%	
Susceptibility profile to the infection in each age group	$\sigma(a)$		Estimates based on fitting the epidemic in China [11]
Age 0-9 years		0.05	
Age 10-19 years		0.05	
Age 20-29 years		0.32	
Age 30-39 years		0.53	
Age 40-49 years		0.65	
Age 50-59 years		0.74	
Age 60-69 years		0.93	
Age 70-79 years		0.88	
Age 80+ years		0.83	
Overall infectious contact rate	β	0.59 contacts per day	Estimate based on fitting the epidemic in China [11]

C. The basic reproduction number R_0

Using the second generation matrix method described by Heffernan *et al.* [12], the basic reproduction number in absence of vaccination is given by

$$R_0 = \sum_{a=1}^{n_{age}} \left[\begin{array}{l} w(a) f_M(a) \left(\dfrac{\beta\delta\sigma(a)}{(\delta+\mu+\xi(a))(\eta_M+\mu+\xi(a))} \right) \\ +w(a) f_S(a) \left(\dfrac{\beta\delta\sigma(a)}{(\delta+\mu+\xi(a))(\eta_{DS}+\mu+\xi(a))} \right) \\ +w(a) f_C(a) \left(\dfrac{\beta\delta\sigma(a)}{(\delta+\mu+\xi(a))(\eta_{DC}+\mu+\xi(a))} \right) \end{array} \right],$$

where $w(a)$ is the proportion of the population in each age group.

Meanwhile, the basic reproduction number in presence of vaccination is given by

$$R_0 = f_M \sum_{a=1}^{n_{age}} w(a) \left(\begin{array}{l} z_{unvac}(a) \left(\dfrac{\beta\delta\sigma(a)}{(\delta+\mu+\xi(a))(\eta_M+\mu+\xi(a))} \right) \\ +z_{vac}(a) \left(\dfrac{\beta\delta\sigma(a)(1+r)(1-VE_S)(1-VE_I)\left((1-VE_{P_2})+VE_{P_2}/f_m\right)}{(\delta+\mu+\xi(a))\left(\eta_M/(1-VE_{P_1})+\mu+\xi(a)\right)} \right) \end{array} \right)$$
$$+f_S \sum_{a=1}^{n_{age}} w(a) \left(z_{unvac}(a) \left(\frac{\beta\delta\sigma(a)}{(\delta+\mu+\xi(a))(\eta_{DS}+\mu+\xi(a))} \right) + z_{vac}(a) \left(\frac{\beta\delta\sigma(a)(1+r)(1-VE_S)(1-VE_I)(1-VE_{P_2})}{(\delta+\mu+\xi(a))(\eta_{DS}+\mu+\xi(a))} \right) \right)$$
$$+f_C \sum_{a=1}^{n_{age}} w(a) \left(z_{unvac}(a) \left(\frac{\beta\delta\sigma(a)}{(\delta+\mu+\xi(a))(\eta_{DC}+\mu+\xi(a))} \right) + z_{vac}(a) \left(\frac{\beta\delta\sigma(a)(1+r)(1-VE_S)(1-VE_I)(1-VE_{P_2})}{(\delta+\mu+\xi(a))(\eta_{DC}+\mu+\xi(a))} \right) \right)$$

where $z_{unvac}(a)$ and $z_{vac}(a)$ are, respectively, the proportions of unvaccinated and vaccinated populations in each age group.

D. Probability of a major outbreak

Based on Whittle's method [13], and by constructing Bailey's ratios [13], the probability of a major outbreak was derived, that is the probability that the fraction of susceptible individuals that become infected is $\geq \gamma$, where γ is a specific chosen level of the final attack rate.

We defined $\rho^U{}_{M,N}(a)$, $\rho^U{}_{S,N}(a)$, and $\rho^U{}_{C,N}(a)$ to be, respectively, Bailey's ratios corresponding to the unvaccinated infectious classes $I_M(a)$, $I_S(a)$, and $I_C(a)$ for each age group. Similarly, we defined $\rho^V{}_{M,N}(a)$, $\rho^V{}_{S,N}(a)$, and $\rho^V{}_{C,N}(a)$ to be, respectively, Bailey's ratios corresponding to the vaccinated infectious classes $I_M^V(a)$, $I_S^V(a)$, and $I_C^V(a)$ for each age group. The number of initial infections is denoted by b.

We define

$$\Omega^U(N) = \sum_{a=1}^{n_{age}} \sum_{i=M,S,C} 1/\rho_{i,N}^U(a)$$

$$\Omega^V(N) = \sum_{a=1}^{n_{age}} \sum_{i=M,S,C} 1/\rho_{i,N}^V(a)$$

.

In the case of no vaccination, the probability of a major outbreak is given by the following three cases:

Case 1

For $\sum_{a=1}^{n_{age}} \sum_{i=M,S,C} \rho_{i,N,U}(a) < N$ and $\sum_{a=1}^{n_{age}} \sum_{i=M,S,C} \rho_{i,N(1-\gamma),U}(a) < N(1-\gamma)$, the probability of a major outbreak lies between $1-\left(\frac{1/\Omega^U(N)}{N}\right)^b$ and $1-\left(\frac{1/\Omega^U(N(1-\gamma))}{N(1-\gamma)}\right)^b$.

Case 2

For $\sum_{a=1}^{n_{age}} \sum_{i=M,S,C} \rho_{i,N,U}(a) < N$ and $\sum_{a=1}^{n_{age}} \sum_{i=M,S,C} \rho_{i,N(1-\gamma),U}(a) > N(1-\gamma)$, the probability of a major outbreak lies between 0 and $1-\left(\frac{1/\Omega^U(N)}{N}\right)^b$.

Case 3

For $\sum_{a=1}^{n_{age}} \sum_{i=M,S,C} \rho_{i,N,U}(a) > N$, the probability of a major outbreak is 0.

Meanwhile, in the case of vaccinating a fraction of the population, the probability of a major outbreak is given by the following three cases:

Case 1

For $\sum_{a=1}^{n_{age}} \sum_{i=M,S,C} \left(\rho_{i,N,U}(a) + \rho_{i,N,V}(a)\right) < N$ and $\sum_{a=1}^{n_{age}} \sum_{i=M,S,C} \left(\rho_{i,N(1-\gamma),U}(a) + \rho_{i,N(1-\gamma),V}(a)\right) < N(1-\gamma)$,

the probability of a major outbreak lies between $1 - \left(\frac{1/\left(\Omega^U(N) + \Omega^V(N)\right)}{N}\right)^b$ and

$$1 - \left(\frac{1/\left(\Omega^U\left(N(1-\gamma)\right) + \Omega^V\left(N(1-\gamma)\right)\right)}{N(1-\gamma)}\right)^b.$$

Case 2

For $\sum_{a=1}^{n_{age}} \sum_{i=M,S,C} \left(\rho_{i,N,U}(a) + \rho_{i,N,V}(a)\right) < N$ and $\sum_{a=1}^{n_{age}} \sum_{i=M,S,C} \left(\rho_{i,N(1-\gamma),U}(a) + \rho_{i,N(1-\gamma),V}(a)\right) > N(1-\gamma)$,

the probability of a major outbreak lies between 0 and $1 - \left(\frac{1/\left(\Omega^U(N) + \Omega^V(N)\right)}{N}\right)^b$.

Case 3

For $\sum_{a=1}^{n_{age}} \sum_{i=M,S,C} \left(\rho_{i,N,U}(a) + \rho_{i,N,V}(a)\right) > N$, the probability of a major outbreak is 0.

Figure S2. Impact of SARS-CoV-2 vaccination on the cumulative number of A) new infections, B) new severe disease cases, C) new critical disease cases, and D) new deaths in the scenario assuming vaccine scale-up to 80% coverage before epidemic onset. Duration of vaccine protection is 10 years. Impact was assessed at $VE_S = 50\%$, $VE_I = 50\%$, $VE_{P_1} = 50\%$, $VE_{P_2} = 50\%$, $VE_S = VE_I = VE_{P_1} = 50\%$.

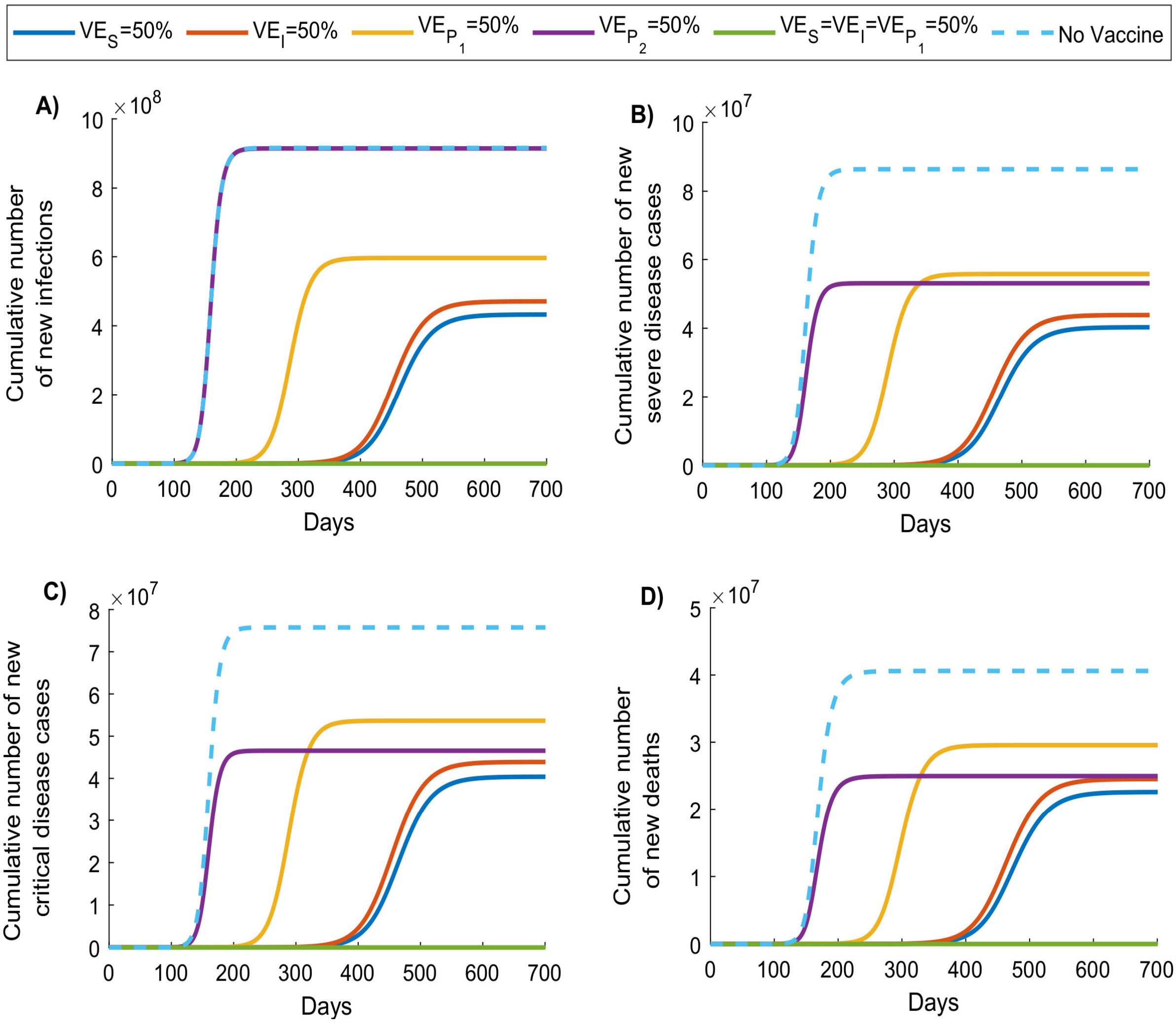

Figure S3. Role of SARS-CoV-2 vaccination in reducing the cumulative number of A) new infections, B) new severe disease cases, C) new critical disease cases, and D) new deaths in the scenario assuming vaccine scale-up to 80% coverage before epidemic onset. Duration of vaccine protection is 10 years. Impact was assessed at $VE_S = 50\%$, $VE_I = 50\%$, $VE_{P_1} = 50\%$, $VE_{P_2} = 50\%$, $VE_S = VE_I = VE_{P_1} = 50\%$.

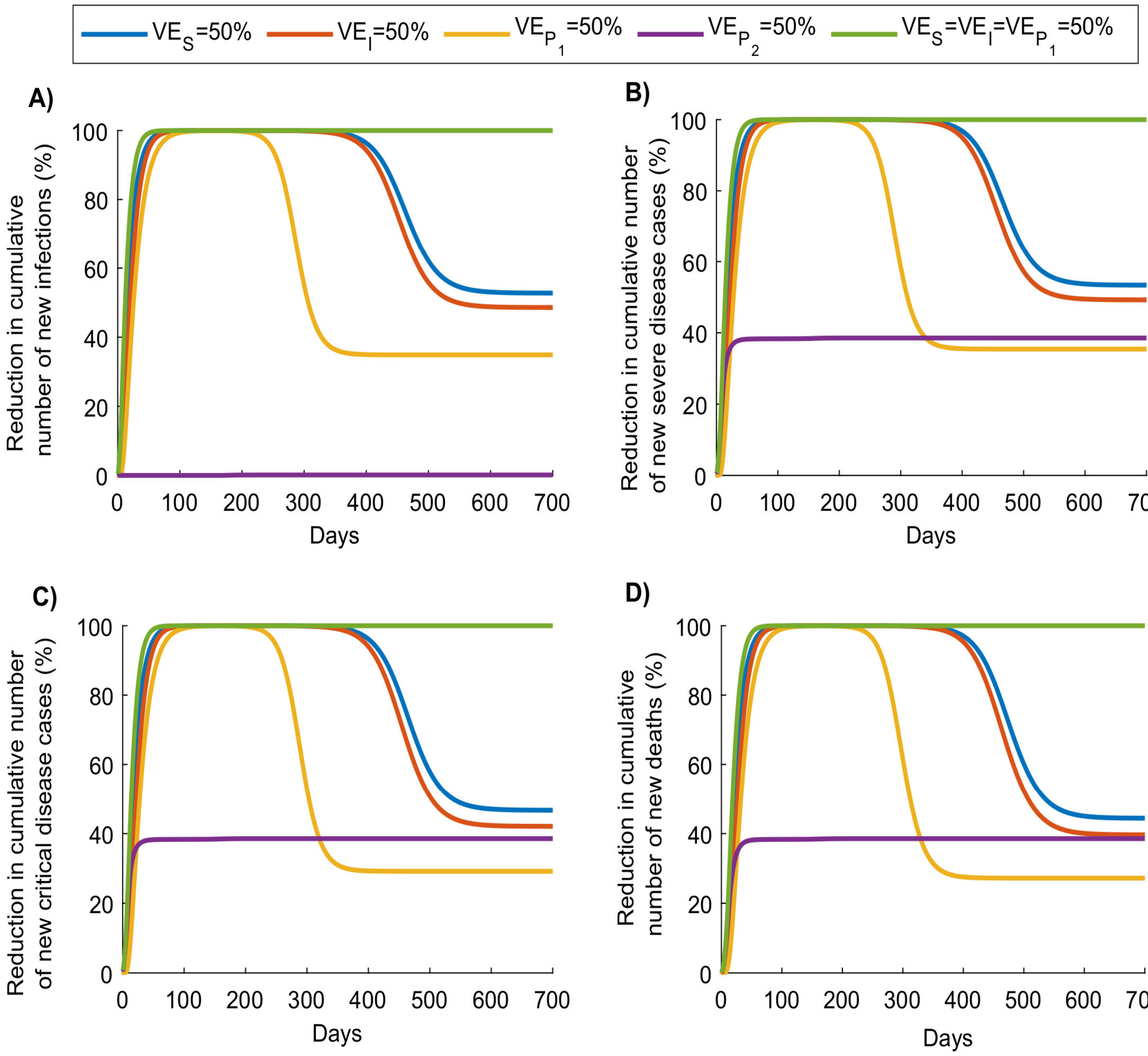

Figure S4. Impact of SARS-CoV-2 vaccination on the cumulative number of A) new infections, B) new severe disease cases, C) new critical disease cases, and D) new deaths in the scenario assuming vaccine introduction during the exponential growth phase of the epidemic, with scale-up to 80% coverage within one month. Duration of vaccine protection is 10 years. Impact was assessed at $VE_S = 50\%$, $VE_I = 50\%$, $VE_{P_1} = 50\%$, $VE_{P_2} = 50\%$, $VE_S = VE_I = VE_{P_1} = 50\%$.

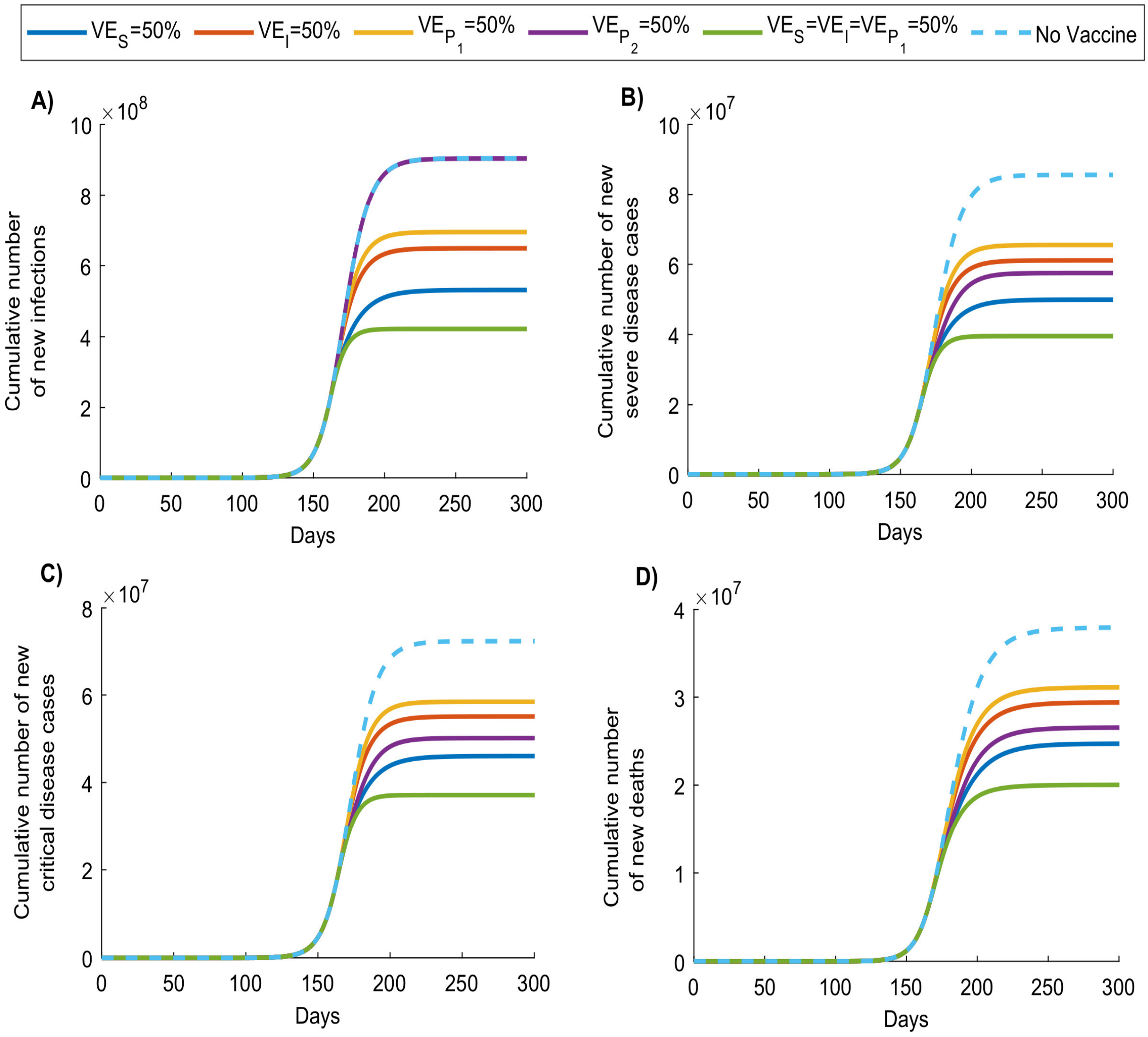

Figure S5. Role of SARS-CoV-2 vaccination in reducing the cumulative number of A) new infections, B) new severe disease cases, C) new critical disease cases, and D) new deaths in the scenario assuming vaccine introduction during the exponential growth phase of the epidemic, with scale-up to 80% coverage within one month. Duration of vaccine protection is 10 years. Impact was assessed at $VE_S = 50\%$, $VE_I = 50\%$, $VE_{P_1} = 50\%$, $VE_{P_2} = 50\%$, $VE_S = VE_I = VE_{P_1} = 50\%$.

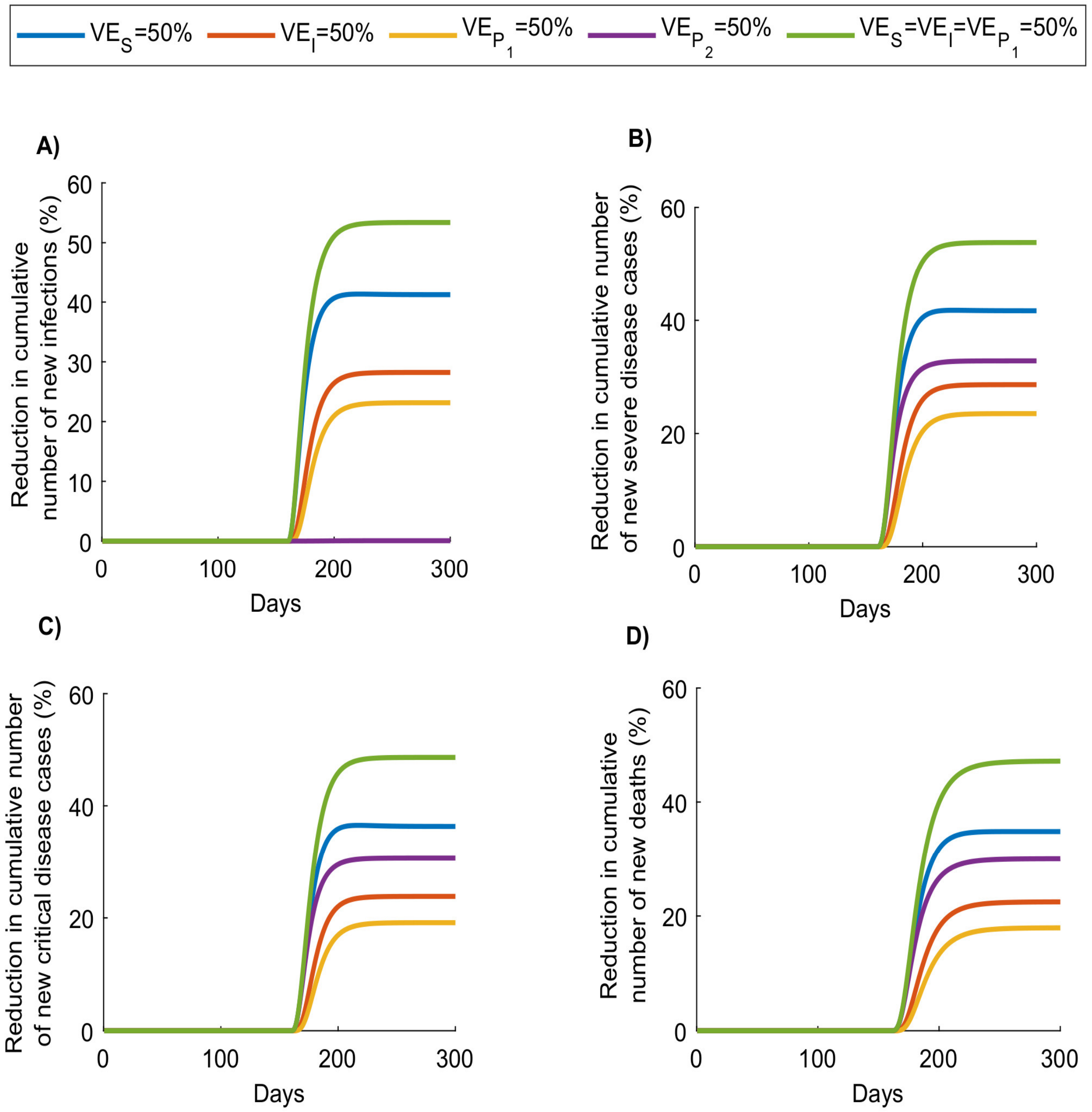

Figure S6. Temporal evolution of SARS-CoV-2 vaccine effectiveness in the scenario assuming vaccine scale-up to 80% coverage before epidemic onset. Number of vaccinations needed to avert A) one new infection, B) one new severe disease case, C) one new critical disease case, and D) one new death, depending on time into the epidemic. Duration of vaccine protection is 10 years. Impact was assessed at $VE_S = 50\%$, $VE_I = 50\%$, $VE_{P_1} = 50\%$, $VE_{P_2} = 50\%$, $VE_S = VE_I = VE_{P_1} = 50\%$. Panel A does not include the result for $VE_{P_2} = 50\%$, as this efficacy has no impact on number of infections—it affects only severe and critical disease and death.

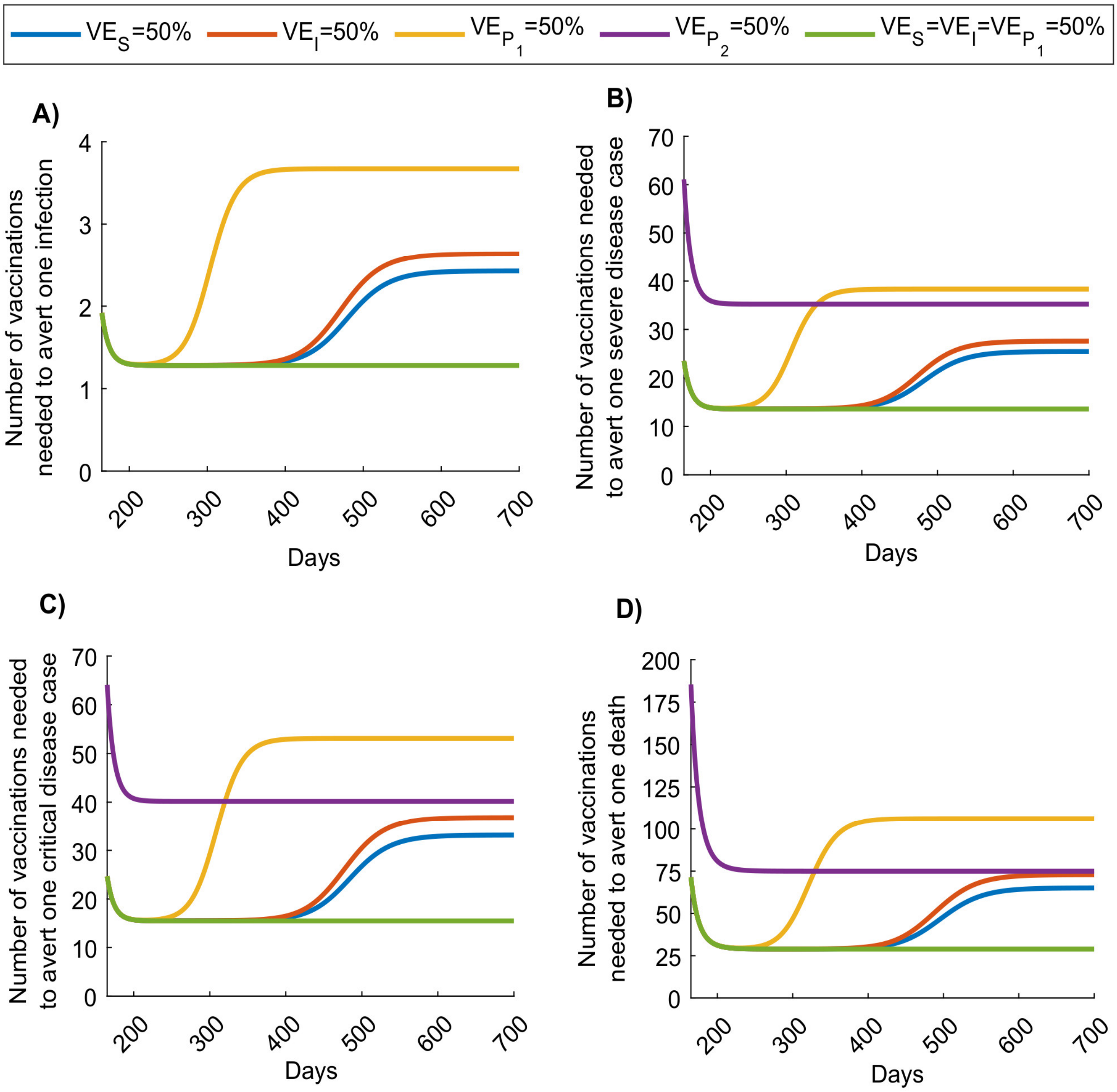

Figure S7. Temporal evolution of SARS-CoV-2 vaccine effectiveness in the scenario assuming vaccine introduction during the exponential growth phase of the epidemic, with scale-up to 80% coverage within one month. Number of vaccinations needed to avert A) one new infection, B) one new severe disease case, C) one new critical disease case, and D) one new death, depending on time into the epidemic. Duration of vaccine protection is 10 years. Impact was assessed at $VE_S = 50\%$, $VE_I = 50\%$, $VE_{P_1} = 50\%$, $VE_{P_2} = 50\%$, $VE_S = VE_I = VE_{P_1} = 50\%$. Panel A does not include the result for $VE_{P_2} = 50\%$, as this efficacy has no impact on number of infections—it affects only severe and critical disease and death.

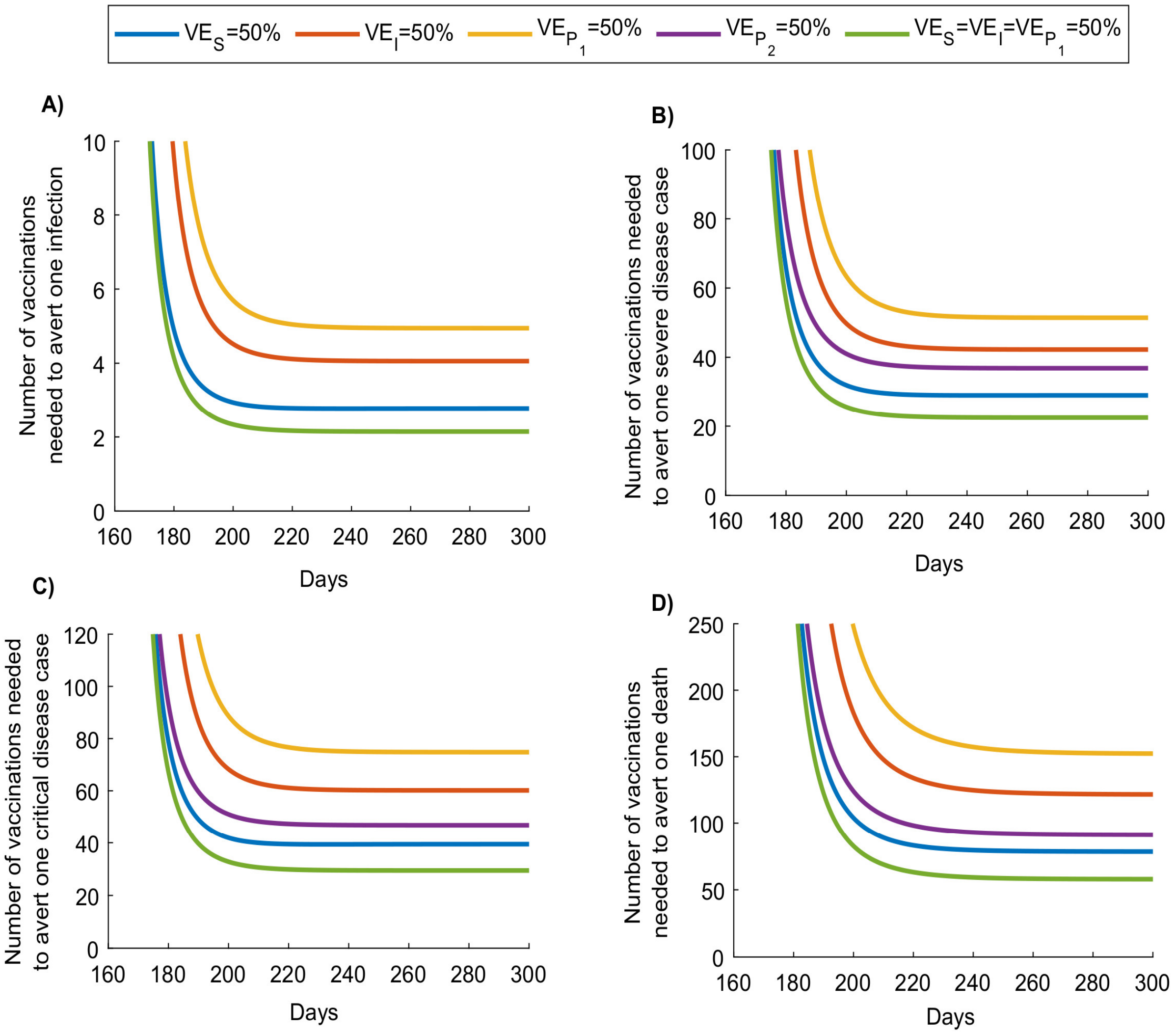

Figure S8. Temporal evolution of effectiveness of age-group prioritization using a SARS-CoV-2 vaccine with *VE*$_s$ of 50%. Number of vaccinations needed to avert A) one infection and B) one death, by prioritizing individuals aged 40-49 or 60-69 years. Scenario assumes vaccine scale-up to 80% coverage before epidemic onset. Duration of vaccine protection is 10 years.

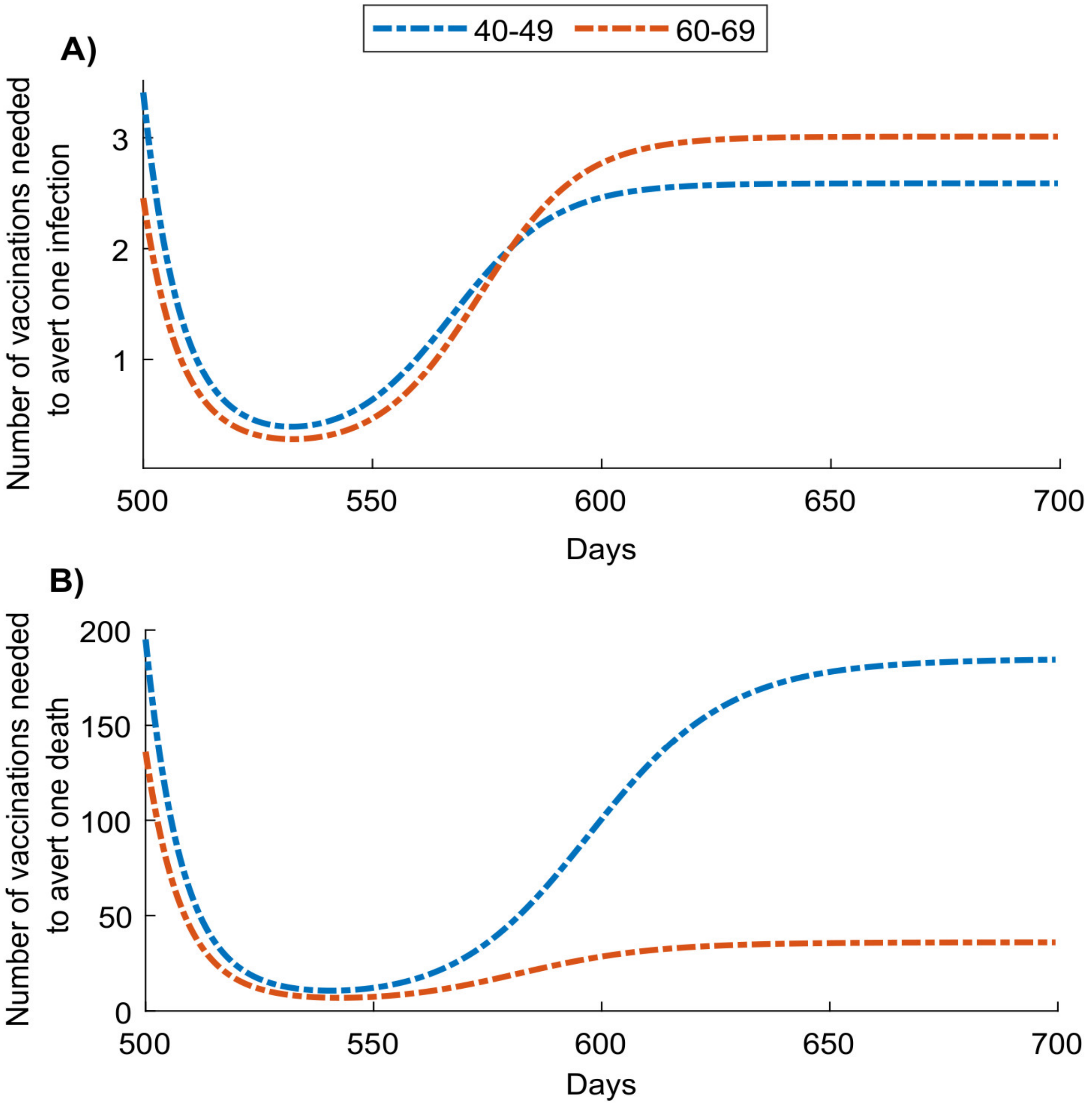

Figure S9. Impact of a social-distancing intervention reducing the contact rate in the population on the cumulative number of new SARS-CoV-2 infections, when introduced to supplement the impact of a vaccine that has 50% efficacy in reducing susceptibility, *VEs*. Scenario assumes vaccine scale-up to 80% coverage before epidemic onset. Duration of vaccine protection is 10 years.

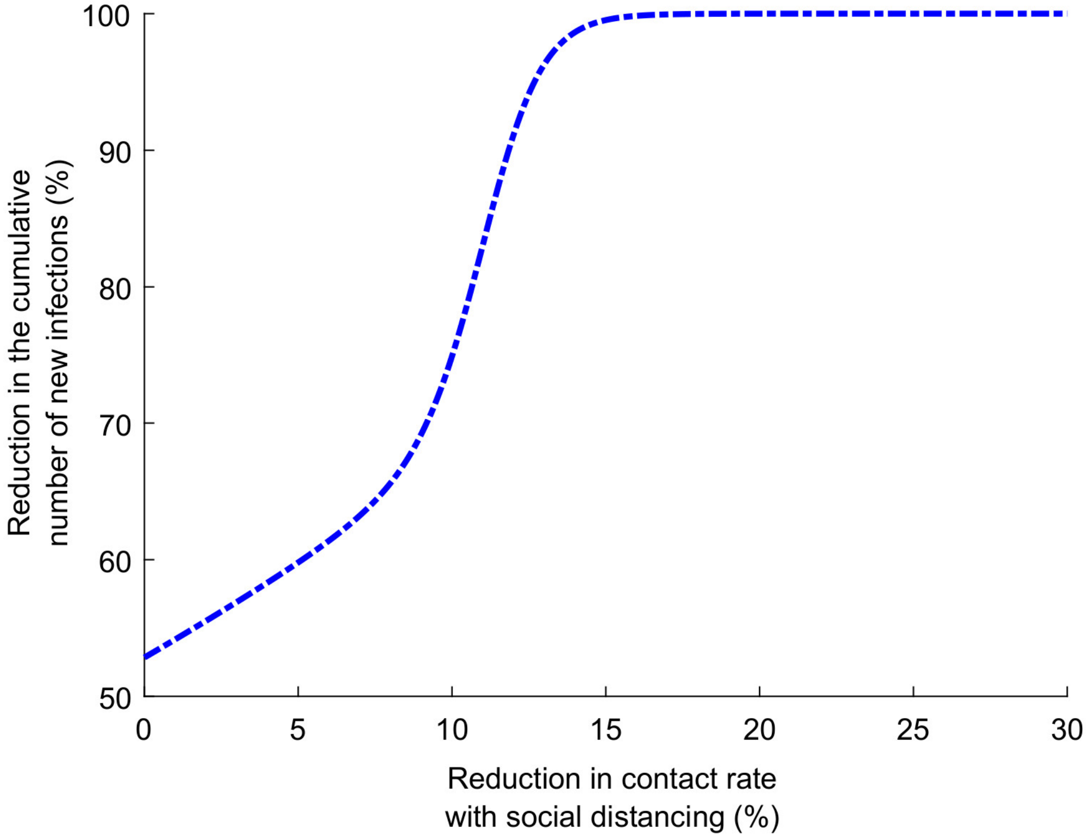

Figure S10. Probability of occurrence of a major outbreak following vaccination. Upper (solid line) and lower (dashed line) bounds of the probability of occurrence of a major outbreak upon virus introduction at varying levels of A) VE_S, B) VE_I, C) VE_{P_1}, and D) $VE_S = VE_I = VE_{P_1}$. Scenario assumes vaccine scale-up to 80% coverage before epidemic onset. Duration of vaccine protection is 10 years. Figure does not include the result for VE_{P_2}, as this efficacy has no impact on probability of occurrence of a major outbreak.

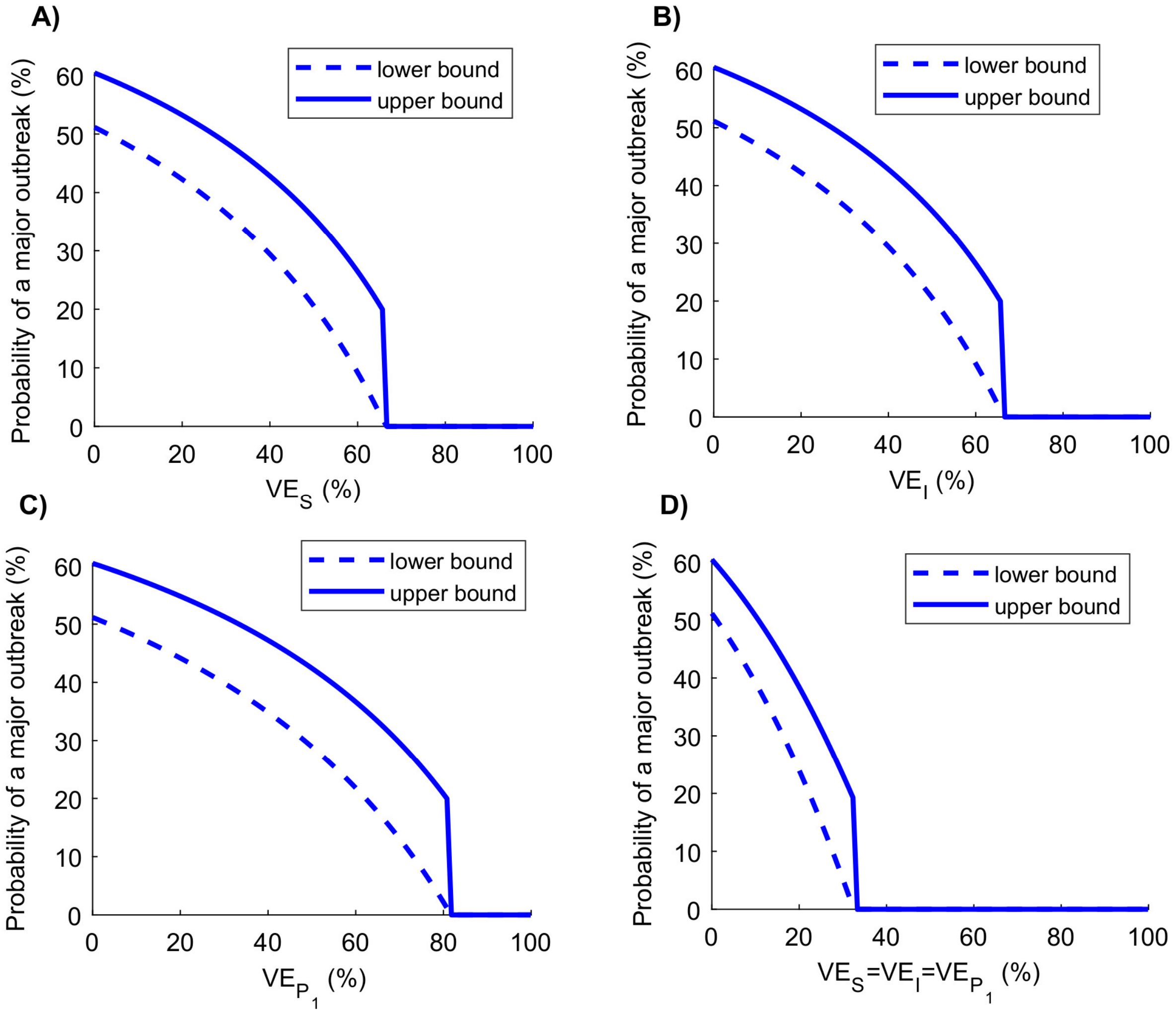

Figure S11. Sensitivity analyses assessing vaccine effectiveness (number of vaccinated persons needed to avert one infection) at A) varying levels of vaccine coverage and B) high levels of assortativeness in age group mixing. Effectiveness is assessed at end of epidemic cycle, that is after the epidemic has reached its peak and declined to a negligible level. Calculations assume vaccine scale-up to the targeted coverage before epidemic onset. Impact was assessed at $VE_S = 50\%$. Duration of vaccine protection is 10 years.

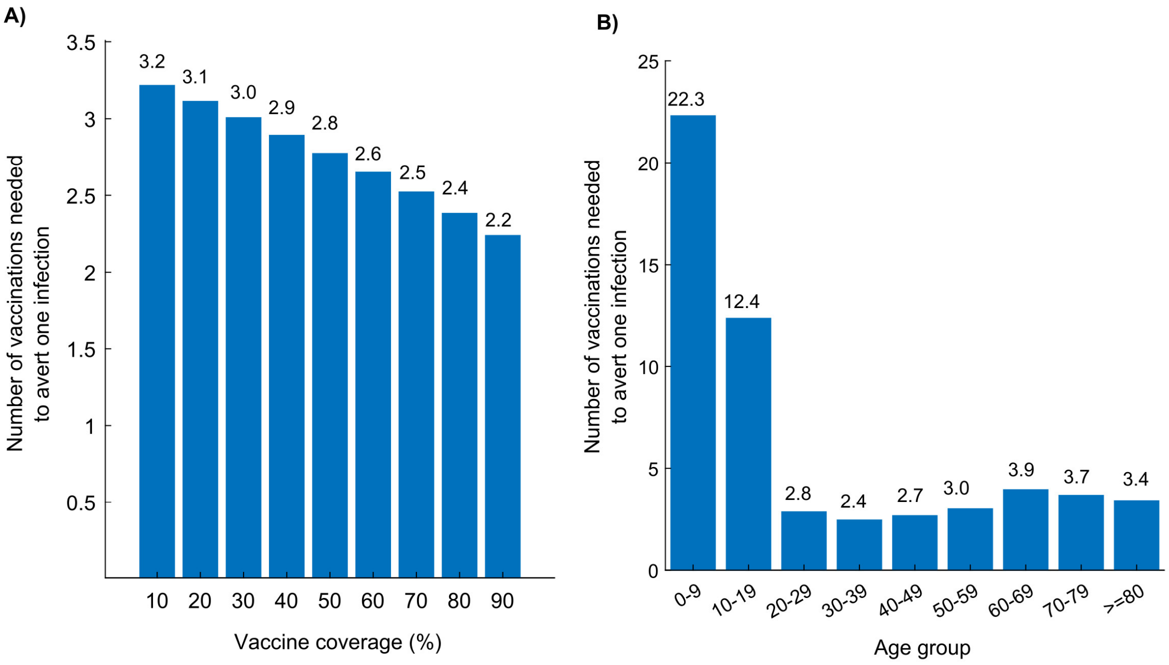

Figure S12. Uncertainty analysis. Model predictions for the mean cumulative number of new infections and associated 95% uncertainty interval (UI) at various levels of vaccine efficacy in reducing susceptibility (VE_S) generated through 500 simulation runs. Scenario assumes vaccine scale-up to 80% coverage before epidemic onset. Duration of vaccine protection is 10 years. The solid black line, dashed lines, and shades show respectively, the mean, 95% uncertainty interval, and individual estimates for the cumulative number of new infections across all 500 uncertainty runs.

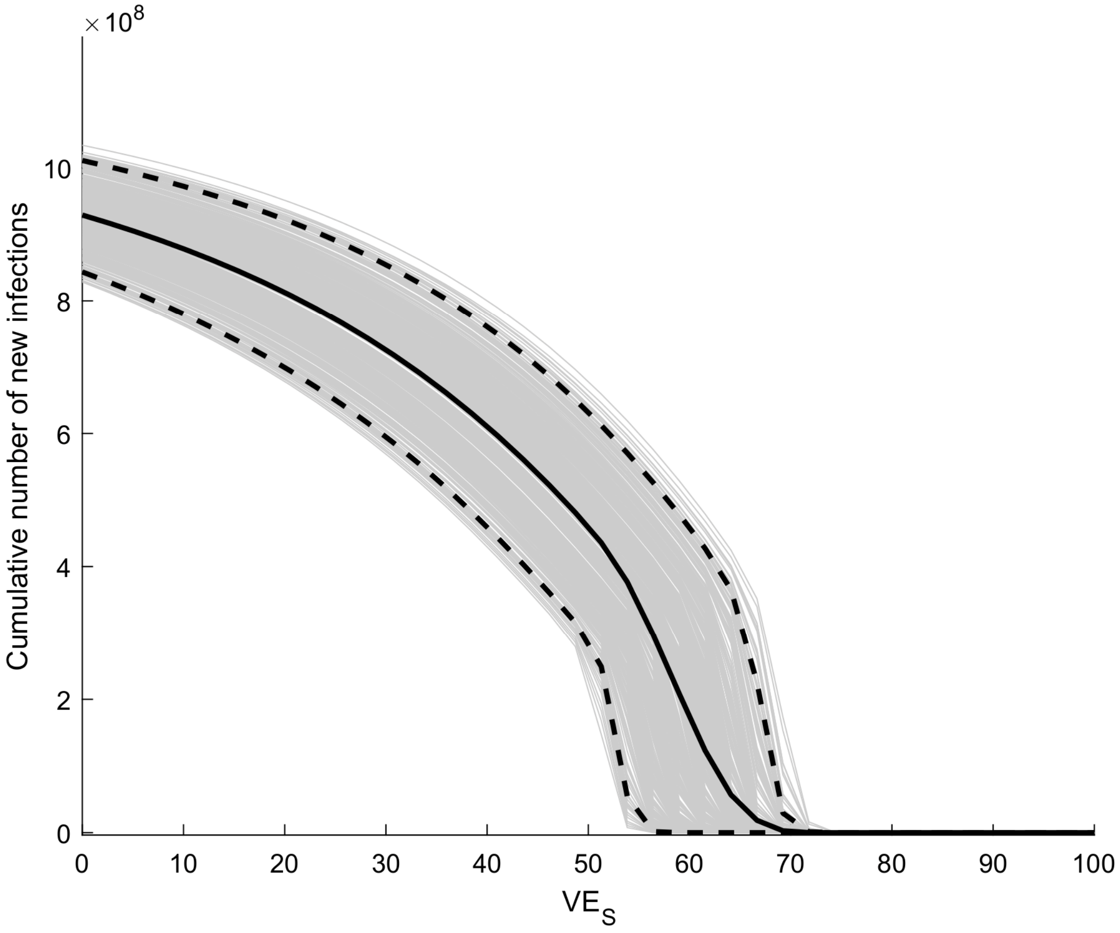

Figure S13. Vaccine effectiveness of age-group prioritization and the reproduction number R_0. Model predictions for SARS CoV-2 attack rate at various levels of R_0, indicating also the impact on R_0 of prioritizing those 60-69 years of age for vaccination or vaccinating all age groups. The blue dashed-dotted line shows the model-predicted attack rate at various levels of R_0. The blue, red, and green stars show, respectively, the model-predicted attack rates in absence of vaccination, by prioritizing vaccination at 80% coverage for those 60-69 years of age, and by extending vaccination at 80% coverage to all age groups. The figure highlights how effectiveness of the vaccine (number of vaccinations needed to avert one infection) depends on the position on the R_0 curve—prioritizing vaccination for any single age group, regardless of that age group, has overall lower effectiveness than extending vaccination to all age groups. The reason is that vaccinating one age group reduces R_0 only marginally, whereas vaccinating all age groups reduces R_0 to an epidemic domain where small reductions in R_0 can have more substantial impact on epidemic size.

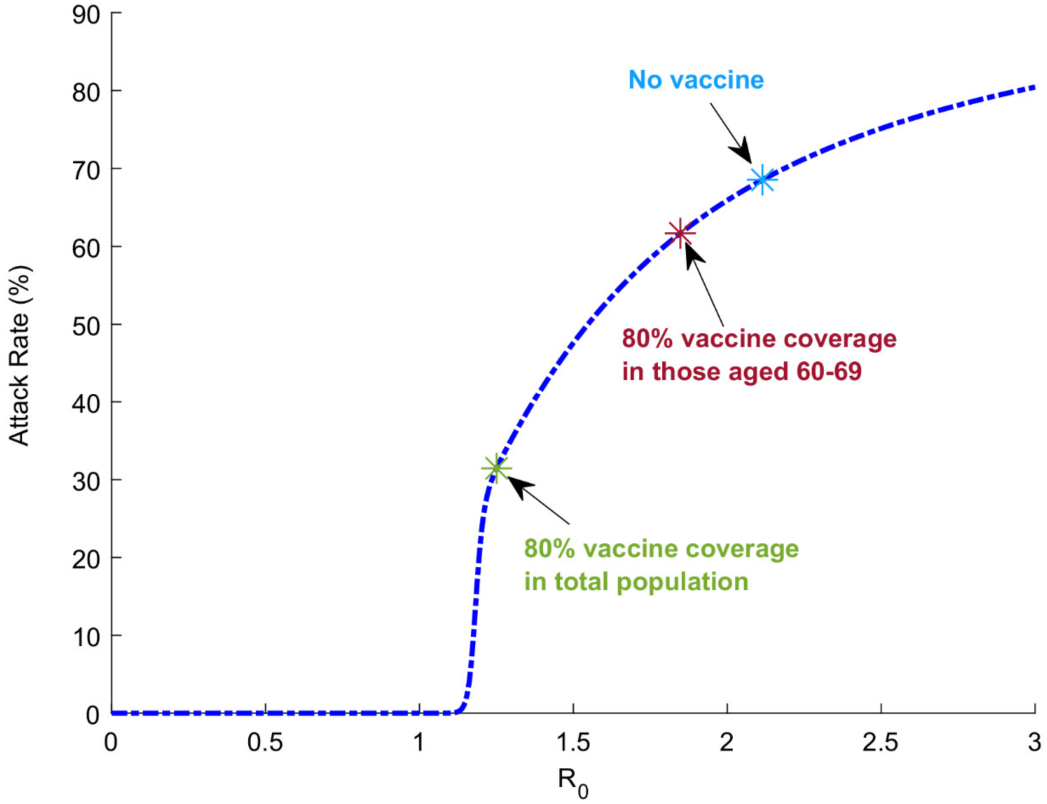

Figure S14. Impact of varying levels of vaccine efficacy in reducing susceptibility, *VE_S*, on the cumulative number of new SARS-CoV-2 infections when the reproduction number R_0 is 3. Scenario assumes vaccine scale-up to 80% coverage before epidemic onset. Duration of vaccine protection is 10 years.

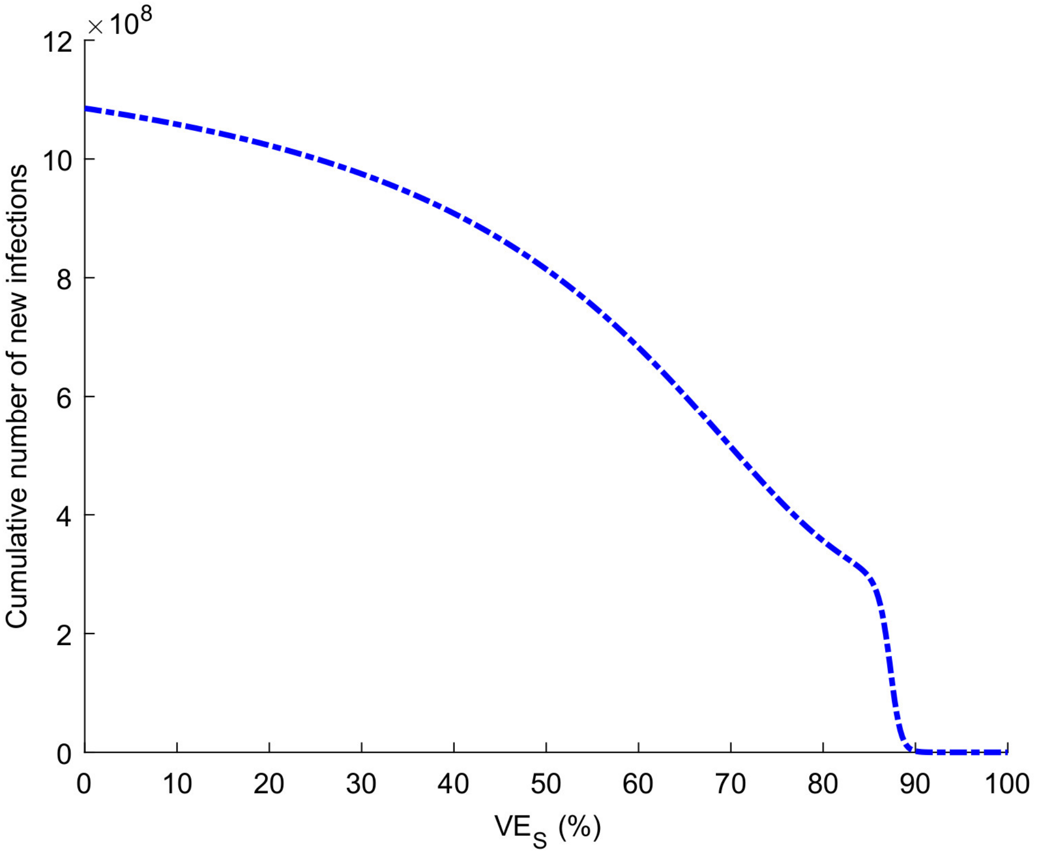

References

[1] Li R, Pei S, Chen B, Song Y, Zhang T, Yang W, et al. Substantial undocumented infection facilitates the rapid dissemination of novel coronavirus (SARS-CoV2). Science. 2020;368:489-93.
[2] Lauer SA, Grantz KH, Bi Q, Jones FK, Zheng Q, Meredith HR, et al. The Incubation Period of Coronavirus Disease 2019 (COVID-19) From Publicly Reported Confirmed Cases: Estimation and Application. Ann Intern Med. 2020;172:577-82.
[3] Zou L, Ruan F, Huang M, Liang L, Huang H, Hong Z, et al. SARS-CoV-2 Viral Load in Upper Respiratory Specimens of Infected Patients. N Engl J Med. 2020;382:1177-9.
[4] Rothe C, Schunk M, Sothmann P, Bretzel G, Froeschl G, Wallrauch C, et al. Transmission of 2019-nCoV Infection from an Asymptomatic Contact in Germany. N Engl J Med. 2020;382:970-1.
[5] World Health Organization. Report of the WHO-China Joint Mission on Coronavirus Disease 2019 (COVID-19). Available from :https://www.who.int/docs/default-source/coronaviruse/who-china-joint-mission-on-covid-19-final-report.pdf. Accessed on March 10, 2020. 2020.
[6] United Nations Department of Economic and Social Affairs Population Dynamics. The 2019 Revision of World Population Prospects. Available from https://population.un.org/wpp/. Accessed on March 1st, 2020. 2020.
[7] Novel Coronavirus Pneumonia Emergency Response Epidemiology Team. [The epidemiological characteristics of an outbreak of 2019 novel coronavirus diseases (COVID-19) in China]. Zhonghua Liu Xing Bing Xue Za Zhi. 2020;41:145-51.
[8] Wu Z, McGoogan JM. Characteristics of and Important Lessons From the Coronavirus Disease 2019 (COVID-19) Outbreak in China: Summary of a Report of 72314 Cases From the Chinese Center for Disease Control and Prevention. JAMA. 2020.
[9] Guan WJ, Ni ZY, Hu Y, Liang WH, Ou CQ, He JX, et al. Clinical Characteristics of Coronavirus Disease 2019 in China. N Engl J Med. 2020.
[10] Huang C, Wang Y, Li X, Ren L, Zhao J, Hu Y, et al. Clinical features of patients infected with 2019 novel coronavirus in Wuhan, China. Lancet. 2020;395:497-506.
[11] Ayoub HH, Chemaitelly H, Mumtaz GR, Seedat S, Awad SF, Makhoul M, et al. Characterizing key attributes of the epidemiology of COVID-19 in China: Model-based estimations. medRxiv. 2020:2020.04.08.20058214.
[12] Heffernan JM, Smith RJ, Wahl LM. Perspectives on the basic reproductive ratio. Journal of the Royal Society Interface. 2005;2:281-93.
[13] Whittle P. The outcome of a stochastic epidemic—a note on Bailey's paper. Biometrika. 1955;42:116-22.
